# SNP arrays: comparing diagnostic yields for four platforms in children with developmental delay

**DOI:** 10.1186/s12920-014-0070-0

**Published:** 2014-12-24

**Authors:** Guylaine D’Amours, Mathieu Langlois, Géraldine Mathonnet, Raouf Fetni, Sonia Nizard, Myriam Srour, Frédérique Tihy, Michael S Phillips, Jacques L Michaud, Emmanuelle Lemyre

**Affiliations:** Service de génétique médicale, CHU Sainte-Justine, Montréal, QC Canada; Centre de recherche, CHU Sainte-Justine, Montréal, QC Canada; Faculté de médecine, Université de Montréal, Montréal, QC Canada; Centre de pharmacogénomique, Institut de cardiologie de Montréal, Montréal, QC Canada; Département de pathologie, CHU Sainte-Justine, Montréal, QC Canada; Pathologie et biologie cellulaire, Université de Montréal, Montréal, QC Canada; Pédiatrie, Université de Montréal, Montréal, QC Canada

**Keywords:** Comparative genomic hybridization (CGH), Congenital abnormalities, Consanguinity, DNA copy number variation (CNV), Intellectual disability, Loss of heterozygosity (LOH), Microarray analysis, Single nucleotide polymorphism (SNP), Uniparental disomy (UPD)

## Abstract

**Background:**

Molecular karyotyping is now the first-tier genetic test for patients affected with unexplained intellectual disability (ID) and/or multiple congenital anomalies (MCA), since it identifies a pathogenic copy number variation (CNV) in 10-14% of them. High-resolution microarrays combining molecular karyotyping and single nucleotide polymorphism (SNP) genotyping were recently introduced to the market. In addition to identifying CNVs, these platforms detect loss of heterozygosity (LOH), which can indicate the presence of a homozygous mutation or uniparental disomy. Since these abnormalities can be associated with ID and/or MCA, their detection is of particular interest for patients whose phenotype remains unexplained. However, the diagnostic yield obtained with these platforms is not confirmed, and the real clinical value of LOH detection has not been established.

**Methods:**

We selected 21 children affected with ID, with or without congenital malformations, for whom standard genetic analyses failed to provide a diagnosis. We performed high-resolution SNP array analysis with four platforms (Affymetrix Genome-Wide Human SNP Array 6.0, Affymetrix Cytogenetics Whole-Genome 2.7 M array, Illumina HumanOmni1-Quad BeadChip, and Illumina HumanCytoSNP-12 DNA Analysis BeadChip) on whole-blood samples obtained from children and their parents to detect pathogenic CNVs and LOHs, and compared the results with those obtained on a moderate resolution array-based comparative genomic hybridization platform (NimbleGen CGX-12 Cytogenetics Array), already used in the clinical setting.

**Results:**

We identified a total of four pathogenic CNVs in three patients, and all arrays successfully detected them. With the SNP arrays, we also identified a LOH containing a gene associated with a recessive disorder consistent with the patient’s phenotype (i.e., an informative LOH) in four children (including two siblings). A homozygous mutation within the informative LOH was found in three of these patients. Therefore, we were able to increase the diagnostic yield from 14.3% to 28.6% as a result of the information provided by LOHs.

**Conclusions:**

This study shows the clinical usefulness of SNP arrays in children with ID, since they successfully detect pathogenic CNVs, identify informative LOHs that can lead to the diagnosis of a recessive disorder. It also highlights some challenges associated with the use of SNP arrays in a clinical laboratory.

**Electronic supplementary material:**

The online version of this article (doi:10.1186/s12920-014-0070-0) contains supplementary material, which is available to authorized users.

## Background

Although intellectual disability (ID) and developmental delay (DD) affect 3% of children [1], etiology remains elusive in close to 50% of cases [2,3], and a diagnosis cannot always be provided to patients and their families, even when a genetic cause is suspected. Many chromosomal aberrations have been associated with syndromic and non-syndromic ID; hence, G-banded karyotyping was, for over 30 years, the first-line genetic analysis for patients with unexplained ID and/or multiple congenital anomalies (MCA). Later, fluorescence *in situ* hybridization was added to the diagnostic arsenal, at first to test patients for whom the clinician suspected a specific microdeletion syndrome, and then to detect subtelomeric rearrangements, which can be found in 3-6% of patients with idiopathic ID [4]. Technological advances in the last decade have changed this testing paradigm, and molecular karyotyping is now the recommended first-tier test for these patients [5-7], as it detects unbalanced chromosomal aberrations at a much higher resolution than conventional karyotype, thus allowing the detection of microdeletions and microduplications without the need to target a specific genomic region.

Initially, molecular karyotyping was performed using BACs in an array-based comparative genomic hybridization (aCGH). Then, oligonucleotides arrays allowed an increase in resolution and a better customization of the tested genomic regions. Array CGH led to the identification of new rare and recurrent submicroscopic pathogenic chromosomal aberrations [8-11], increasing the diagnostic yield in patients with unexplained ID and/or MCA from 3.7% (G-banded karyotype) [12] to 10-14% (molecular karyotype) [6,13,14]. Conversely, an important number of chromosomal aberrations that are thought to be benign, or for which clinical significance currently cannot be determined, are being identified [13], requiring cytogenetics laboratories to store and share genotype-phenotype data in order to provide the most accurate clinical interpretation for each result.

Although high-resolution SNP arrays have been used extensively in genome-wide association studies [15-17], cytogenetics laboratories have only recently begun using them in the clinical setting, and available reports are restricted to their use for copy number variation (CNV) detection [18-20]. However, the SNP genotyping information provided by these arrays also allows loss of heterozygosity (LOH) detection, which can be the result of a deletion, autozygosity (identity-by-descent) or uniparental disomy (UPD). In a clinical setting, this is of interest because autozygous regions may either: 1) harbour disease-causing homozygous mutations, as illustrated by the higher rate of MCA, ID, and other serious medical conditions in consanguineous families [21-23]; or 2) signal UPD, whether segmental or affecting a whole chromosome, which can cause syndromic conditions when it involves imprinted genes [24]. This extends the scope of molecular karyotyping beyond chromosomal aberrations detection, to include the detection of pathogenic genomic abnormalities that would usually require separate molecular analyses.

In this study, we test children affected with ID/DD on four high-resolution SNP arrays, and compare the results with the moderate-resolution oligo-array currently used in our laboratory. Our goal is to evaluate, in the clinical setting, the performance and workload of each platform, and to determine whether there is an additional value in using one of these arrays to test patients with ID and/or MCA. We compare the proportion of pathogenic and benign CNVs, as well as variants of uncertain clinical significance (VOUS); examine LOH larger than 5 Mb; and compare the characteristics of each vendor’s provided visualization software.

## Methods

### Patients

Twenty-one patients, between the age of six months and 17 years old, were recruited consecutively upon evaluation by a clinical geneticist at the Centre Hospitalier Universitaire Sainte-Justine. Inclusion criteria were as follow: 1) the child presented DD (n = 13), ID (n = 5), or both (n = 3); 2) the geneticist ordered aCGH as part of the clinical investigation; and 3) both biological parents were available for testing. A subset of children also had autism (n = 3), or pervasive developmental delay (n = 2), or presented dysmorphic features (n = 3), malformation(s) (n = 3), or both (n = 7). Phenotypic information was collected both for the children and their parents. Phenotypic details are available in Table 1. Patients were excluded from the study if a single-gene disorder seemed most probable, or if a chromosomal rearrangement (balanced or unbalanced) had previously been detected. Children older than 16 years old and parents’ written informed consent were obtained for inclusion in the research protocol and publication of individual clinical details, data and results. For younger children, parental written informed consent was obtained for the same purpose, as well as child’s assent when possible. This study was approved by the CHU Sainte-Justine’s Research Ethics Board.

**Table 1:** **Phenotype information of patients included in the study**

**Patient**	**Sex**	**Age** ^**a**^	**Diagnosis** ^**b**^	**Dysmorphism**	**Malformation(s)**	**Other**
1.1	F	12 y	ID	Yes	Multiple: multicystic dysplastic kidney (left), ventriculomegaly, temporal white matter loss, pectus excavatum	Deafness, myopia, nystagmus, dysphagia, mild scoliosis
3.2	M	6 mo	Growth delay	Yes	Cerebral: microcephaly, gyral simplification, myelination delay	Spastic quadriparesis
6.3	M	11 y	PDD, mild ID	Yes	Mild: pectus excavatum	Café au lait spots. Mother: school difficulties, epilepsy, murmur, arthrosis
10.4	M	7 y	Global DD, PDD	No	No	Hypotonia, café au lait spots, pes planus
11.4	M	13 y	Global DD	No	No	Simple febrile convulsions, hypotonia, diminished deep tendon reflexes
14.5	F	14 y	Mild ID, growth delay	Yes	Multiple: microcephaly, thick corpus callosum, malocclusion, filum terminale lipoma, sacral agenesis	Feeding difficulties
17.6	M	3 y	DD, autism	No	No	-
20.7	M	18 mo	Speech delay	Yes	Craniofacial: trigonocephaly, labiopalatine cleft	Heterochromia, facial asymmetry
23.8	M	13 y	DD, mild ID	No	Multiple: cerebellar atrophy, malocclusion, valgus feet	Ataxia
26.9	M	5 y	Global DD, mild ID	Yes	No	Obesity, ataxia, buccolingual dyspraxia, increased lactates (blood & LCR)
29.10	M	17 y	ID, autism	Yes	No	Obesity, retinitis pigmentosa
32.11	M	2 y	DD	Yes	Cerebral: microcephaly, pons size slightly reduced, 4th ventricle size slightly increased	Short stature, epilepsy, gastroesophageal reflux
35.12	M	17 mo	Global DD	Yes	Multiple: optic atrophy, thin corpus callosum, cerebral atrophy, myelination delay	Epilepsy, hypotonia, mild limb spasticity
38.13	F	6 y	ID	No	No	-
41.14	M	22 mo	Global DD	Yes	No	Ligament hypermobility. Father: learning disability
44.15	M	4 y	DD, autism	No	Craniofacial: submucosal palatine cleft	Velopharyngeal insufficiency
47.16	M	3 y	Global DD	No	Pulmonary valve stenosis	Gastroesophageal reflux
50.17	F	10 y	Global DD	No	No	Epilepsy
53.18	M	5 y	Global DD	No	No	Tall stature
56.19	F	11 y	Mild ID	No	No	Strabismus
59.20	F	34 mo	Atypical development	No	No	Mild facial asymmetry

^a^y: years, mo: months.

^b^ID: intellectual disability, PDD: pervasive developmental disorder, DD: developmental delay.

### Samples

Peripheral blood samples were drawn from each individual and kept at −20°C until DNA purification. Purified genomic DNA was obtained using the QIAamp DNA Blood Midi Kit (QIAGEN, Mississauga, ON, Canada) according to manufacturer’s instructions and stored at 4°C. Absorbance measurements were performed with a NanoDrop 1000 spectrophotometer (Thermo Scientific, Wilmington, DE, USA) to verify DNA purity and adjust DNA concentration. Genomic DNA length was controlled for each DNA sample by running 50 ng of DNA diluted in 10 uL of TE buffer (QIAGEN) on a 1.2% agarose E-Gel (Invitrogen, Carlsbad, CA, USA).

### Array genomic hybridization

DNA samples were sent to an external laboratory (Centre de Pharmacogénomique Beaulieu-Saucier, Montréal, Qc, Canada) for array genomic hybridization, which was performed according to manufacturer’s instruction with the following four arrays: Affymetrix Genome-Wide Human SNP Array 6.0 (SNP 6.0) and Cytogenetics Whole-Genome 2.7 M array (2.7 M) (Affymetrix, Santa Clara, CA, USA), and Illumina HumanOmni1-Quad BeadChip (Omni1) and HumanCytoSNP-12 DNA Analysis BeadChip (CytoSNP) (Illumina, San Diego, CA, USA). Samples were also analysed with the NimbleGen CGX-12 Cytogenetics Array (CGX-12) (Roche NimbleGen, Madison, WI, USA) for all patients, as previously described [25]. This CGH array contains 135 000 oligonucleotides targeting 675 genes and more than 200 important cytogenetic regions with an average spacing of 65–75 kb throughout the genome, and 10 kb in targeted regions. Parents were tested with this array only if required for CNV interpretation (n = 2).

### Visualization software settings

CNVs and LOH were visualized in the software provided by each vendor: Affymetrix Chromosome Analysis Suite 1.1 (ChAS) for SNP 6.0 and 2.7 M, Illumina KaryoStudio 1.3.1 for Omni1 and CytoSNP, and Signature Genomics Genoglyphix for CGX-12. For CGX-12, we used the thresholds currently in place in the clinical laboratory, which are as follows: imbalances spanning a minimum of five oligonucleotides, with a mean log_2_ ratio above 0.4 for gains or below −0.7 for losses. For Affymetrix and Illumina arrays, confidence was set at 80% for 2.7 M (SNP 6.0 does not offer a confidence statistic) and 50% for the Illumina arrays. In ChAS, we used the following configuration: Smoothing enabled and Joining enabled (number of markers = 1). There is no such custom configuration offered in KaryoStudio. For CNV calls visualization, we used two different sets of thresholds, a standard set and an optimized set (Table 2). The standard set used parameters recommended by the manufacturer, and we anticipated that this approach would lead to a large number of CNV calls. To reduce the number of CNV calls to interpret, we explored how we could use the various filters available in both visualization softwares. In addition, we found that using a standard size threshold of 50 kb would miss smaller pathogenic CNVs as those found in our lab and in the literature [26,27]. Our goals with the use of optimized thresholds were: 1) to reduce the total number of CNV calls to interpret, while preserving potentially pathogenic alterations; 2) decrease the size threshold in order to detect small pathogenic CNVs; and 3) remove known benign CNVs. We accomplished this by: 1) increasing thresholds in genomic regions exempt of genes of known clinical relevance; 2) decreasing thresholds in regions with genes of known clinical relevance; and 3) filtering out known benign CNVs. We used the adjustable parameters in both softwares to perform steps 1 and 2, by applying different thresholds to regions with genes catalogued in OMIM Morbid Map (Online Mendelian Inheritance in Man Morbid Map, Feb 2009 hg19 assembly, accessed October 28, 2010) and regions without such genes. For the third step, we took advantage of a feature available in ChAS, which allows masking CNV calls with a certain % of overlap with specific genomic regions, to filter out CNV calls without genes of known clinical relevance that also had a 100% overlap with CNVs reported in DGV (Database of Genomic Variants, Build 36(hg18), released July 2009). In KaryoStudio, this filter was not used since it was not possible to apply it only in certain regions, excluding those overlapping OMIM genes. Both sets of thresholds are detailed in Table 2, as well as thresholds used for long contiguous stretches of homozygosity (LCSH) visualization.

**Table 2:** **ChAS and KaryoStudio thresholds used for CNV and LCSH visualization**

	**Parameter**		**SNP 6.0**	**2.7 M**	**Omni1**	**CytoSNP**
CNV (standard)	Size		≥50 Kb	≥50 Kb	≥50 Kb	≥50 Kb
	Marker count^a^		≥25	≥25	≥7	≥7
CNV (optimized)	Size	Inside OMIM^b^	≥10 Kb	≥10 Kb (loss), ≥50 Kb (gain)	≥10 Kb	≥10 Kb
		Outside OMIM	≥100 Kb	≥200 Kb	≥100 Kb	≥100 Kb
	Marker count	Inside OMIM	≥10	≥10	≥5	≥5
		Outside OMIM	≥25	≥25	≥7	≥7
	DGV^c^	Outside OMIM	Removed	Removed	N/A	N/A
LCSH	Size		≥5 Mb	≥5 Mb	≥5 Mb	≥5 Mb
	Marker count		≥100	≥100	≥20	≥20

*N/A*: non-applicable.

^a^Abnormal markers within the CNV or LCSH segment.

^b^ *OMIM* : Regions containing genes of known clinical relevance from the NCBI *Online Mendelian Inheritance in Man* Morbid Map (February 2009 hg19 assembly, accessed October 28, 2010).

^c^CNV completely overlapping with a CNV reported in *Database of Genomic Variants* (NetAffx Build 30.2).

### CNV interpretation

Inheritance for detected CNVs was determined by visualizing a child and his parents’ results side by side in the software, assigning an inherited status to a child’s CNV call if there was ≥ 80% overlap with a parental CNV call. Since microarray analysis alone does not allow chromosomal position analysis of the detected CNVs, apparently *de novo* CNVs could in fact be inherited from a parent through an insertional translocation, although these rearrangements are rare events (incidence of insertional translocations detected by microarrays was estimated at 1:3380 to 1:5200 in the general population by Neill *et al.* [28] and 1 in 500 individuals referred for clinical CGH analysis by Kang *et al.* [29]). Because we are interested in the copy number state, which in that case would indeed be *de novo,* and for conciseness purposes, we will refer to all apparently *de novo* CNVs as “*de novo* CNVs”. Due to the high number of detected CNVs by some platforms using standard thresholds (see Table 2), we selected CNV calls with a high probability of being pathogenic for the analysis with optimized thresholds: *de novo* CNV calls, homozygous CNV calls inherited from heterozygous parents, and maternally inherited CNV calls on chromosome X found in boys. There were only 14 CNV calls in the latter two categories, which are later referred to as *de novo* CNV calls. Therefore, inherited CNV calls do not include these. Each *de novo* CNV call was then classified into one of three categories, based on its clinical significance: benign, pathogenic, or VOUS. Clinical significance was established by comparing each unbalanced genomic region to information from public & private databases (DGV, OMIM, and Genoglyphix Chromosome Aberration Database, GCAD [30]). CNVs reported in DGV or overlapping segmental duplications were considered benign. For CGX-12, CNV calls previously reported in at least two healthy individuals from our internal database or GCAD were also considered benign. CNV calls larger than 5 Mb or involving genomic regions of known clinical relevance (OMIM Morbid Map) compatible with the patient’s phenotype were classified as pathogenic. Remaining CNV calls were classified as VOUS.

### LOH interpretation

For LOH interpretation, as homozygous regions can indicate hemizygosity, autozygosity (identity-by-descent), or UPD, which can suggest a recessive or imprinting disorder that would require confirmation by molecular analyses, we excluded LOHs resulting from a deletion and only interpreted LCSHs. For UPD detection, we looked for autosomal LCSHs spanning a whole chromosome, which would reveal uniparental isodisomy (iUPD), and autosomal LCSHs larger than 10 Mb, which could indicate a mix of uniparental isodisomy and heterodisomy, or segmental UPD [31]. For UPD detection purposes, we excluded large LCSHs present in patients who also had large LCSHs (>10 Mb) in multiple chromosomes, as this is suggestive of parental consanguinity rather than UPD. Genotyping information was extracted and analyzed in Excel for all trios, to confirm parental origin of LCSHs in all patients, and to identify genotyping discordances between parents and child, which would indicate UPD (isodisomy or heterodisomy). We calculated percentage of homozygosity as described by Kearney *et al.* [32], using the following equation for each patient: total length of LCSHs in Mb, divided by total autosomal length in Mb. To identify LCSHs potentially indicative of a homozygous mutation, we studied LCSHs larger than 5 Mb, and compared gene content with information from the OMIM database. We considered a LCSH informative if it contained a gene associated with a recessive disorder consistent with the patient’s phenotype. Other LCSHs were considered uninformative.

## Results

The goal of this study was to evaluate, in a clinical context, the diagnostic performance of SNP arrays combining CNV detection and SNP genotyping information. Twenty-one patients were tested on four different platforms from two manufacturers (Affymetrix SNP 6.0 and 2.7 M arrays; and Illumina Omni1 and CytoSNP arrays), and their results were compared with those obtained on the NimbleGen CGX-12 array, currently in use at our laboratory. In order to do this, we optimized the CNV detection thresholds for clinical application, and interpreted all *de novo* CNV calls, as well as regions of homozygosity that could be of clinical interest. We also compared each visualization software’s features, ease of use, and efficiency in a clinical setting.

### Detected CNVs: standard thresholds

Using standard thresholds (Table 2), SNP 6.0 and 2.7 M from Affymetrix respectively detected 503 and 368 CNVs for all 21 patients, while Omni1 and CytoSNP from Illumina detected 197 and 30 CNVs, respectively (Additional file 1: Table S1). The ratio of copy number gains versus copy number losses was close to 1:1 for Omni1 and CytoSNP (89:108, and 16:14, respectively), while it was roughly 6:1 for SNP 6.0 and 2.7 M (430:73, and 314:54, respectively). The proportion of *de novo* CNVs calls varied from 17.3% (34/197) for Omni1 to 47.8% (176/368) for 2.7 M. CytoSNP called the lowest number of CNVs per patient, with an average of 1 (range 0–5), while SNP 6.0 called the highest, with an average of 24 CNVs per patient (range 14–37). Overall, both Affymetrix arrays called a very high number of CNVs, the majority (85%) being gains, while Illumina arrays called less CNVs, and gains and losses were more balanced.

### Detected CNVs: optimized thresholds

The approach with optimized thresholds resulted in an important reduction in the total number of CNV calls by the Affymetrix arrays, while numbers remained relatively similar for both Illumina arrays (Additional file 1: Table S1 and Additional file 2: Table S2). The proportion of *de novo* CNV calls also remained similar for the Illumina arrays, but increased from 21.7% (109/503) to 55.6% (139/250) for SNP 6.0, and from 47.8% (176/503) to 69.4% (150/216) for 2.7 M. These differences in the overall number and the proportion of *de novo* CNV calls were expected, since we removed benign CNVs reported in DGV (which would mostly be inherited) for the Affymetrix arrays, but we were technically unable to do so with KaryoStudio. The ratio of gains vs losses decreased from 6:1 to about 4:1 for Affymetrix arrays, while it slightly increased from 1:1 to 1.7:1 for CytoSNP, and remained unchanged at around 1:1 for Omni1. The average number of CNV calls per patient was cut in half for Affymetrix arrays: from 24 to 12 for SNP 6.0 (range 4–30) and from 18 to 10 for 2.7 M (range 1–32). There was a slight decrease in the average number of CNV calls per patient for Omni1, which went from 10 to 9 (range 5–13) and there was no change for CytoSNP (1 CNV/patient, range 0–5). In comparison, CGX-12 detected 58 CNVs for all 21 patients, for an average of 3 CNV calls per patient (range 0–7), and a ratio of gains vs losses of 0.9:1 (27:31). Inherited vs *de novo* proportions cannot be compared with the CGX-12 array because parents were only tested if required for CNV interpretation. Optimized thresholds resulted, as expected, in a reduction of the gains vs losses ratio and of the overall number of CNVs detected by the Affymetrix arrays, while it had almost no impact on the Illumina arrays.

### CNV validation

Using an alternate microarray is a recognized method of confirmation for detected CNVs [7,33]. In order to confirm CNV calls, we compared results obtained with the optimized thresholds from each array, and considered a CNV validated if it was detected on at least two platforms with > 50% overlap. The list of confirmed CNVs is available in Additional file 3: Table S3. The confirmation rate was very low for most arrays except CytoSNP, for which 96.0% of the CNV calls were also seen on at least another array (Table 3). In comparison, NimbleGen CGX-12 array had a confirmation rate of 46.6%. There was an important difference in the confirmation rate of gains vs losses on the SNP 6.0 and 2.7 M arrays (13.9% vs 40.8%, and 7.6% vs 25.0%, respectively), while Omni1, CytoSNP and CGX-12 arrays showed a similar confirmation rate for gains and losses. Also, the confirmation rate was higher for inherited CNV calls than for *de novo* CNV calls for SNP 6.0, 2.7 M and CytoSNP (27.9% vs 12.2%, 19.7% vs 7.3%, and 100% vs 87.5%, respectively) while it was similar for Omni1 (30.0% of inherited vs 34.3% of *de novo*). In order to verify that the unconfirmed CNV calls were not due to an inadequate detection algorithm or lack of coverage on the other arrays, we also examined, on all arrays, the signal of unconfirmed *de novo* CNV calls that were later classified as pathogenic or VOUS. None of those CNV calls had a detectable signal on another array, and the majority (80.9%) had adequate coverage on at least one additional array (data not shown). In summary, very few gains detected on the Affymetrix arrays were confirmed, while there was no such bias with the Illumina or the NimbleGen arrays. Also, the confirmation rate of all arrays, except Omni1, was higher for inherited CNV calls than for *de novo* CNV calls.

**Table 3:** **Number of confirmed CNVs and confirmation rate for each tested array**

**Confirmed CNVs**	**SNP 6.0**	**2.7 M**	**Omni1**	**CytoSNP**	**CGX-12**	**All arrays**
Total number (%^a^)	48 (19.2%)	24 (11.1%)	57 (30.8%)	24 (96.0%)	27 (46.6%)	180 (72.0%)
Gains (%^b^)	28 (13.9%)	13 (7.6%)	30 (38.5%)	15 (93.8%)	14 (51.9%)	100 (20.2%)
Losses (%^c^)	20 (40.8%)	11 (25.0%)	27 (25.2%)	9 (100.0%)	13 (41.9%)	80 (33.3%)
De novo (%^d^)	17 (12.2%)	11 (7.3%)	12 (34.3%)	7 (87.5%)	25^e^ (44.6%)	72 (51.8%)
Inherited (%^f^)	31 (27.9%)	13 (19.7%)	45 (30.0%)	17 (100.0%)	2 (100.0%)	108 (77.7%)

^a^Confirmation rate = number of confirmed CNVs divided by number of detected CNVs by each array, expressed in %.

^b^Confirmation rate = number of confirmed gains divided by number of detected gains by each array, expressed in %.

^c^Confirmation rate = number of confirmed losses divided by number of detected losses by each array, expressed in %.

^d^Confirmation rate = number of confirmed *de novo* CNVs divided by number of detected *de novo* CNVs by each array, expressed in %.

^e^De novo or inheritance unknown (parents not systematically tested).

^f^Confirmation rate = number of confirmed inherited CNVs divided by number of detected inherited CNVs by each array, expressed in %.

### Clinical significance of detected *de novo* CNVs

We interpreted all detected *de novo* CNVs (confirmed or not) on each platform individually, using the manufacturer’s accompanying visualization software, and assigned each CNV call one of three categories: pathogenic, benign, or VOUS. In order to have a true assessment of each array’s performance in a clinical setting, we classified each CNV call independently from the information gained on the other arrays. There was a total of 332 *de novo* CNVs detected on any of the four tested arrays for all 21 patients (Additional file 2: Table S2). CytoSNP detected 8 *de novo* CNVs, Omni1 detected 35, SNP 6.0 detected 139 and 2.7 M detected 150 (Table 4). Seventeen of these were false positives that should not have been included in the *de novo* CNVs group. These were either falsely called because ChAS joined two small segments separated by the uncovered centromere, which falsely increased their size above the threshold, or they were falsely assigned to the *de novo* category — when in fact they were inherited — because the software did not call the parental CNV despite a clear signal.

**Table 4:** **Clinical significance of detected de novo CNVs**

**De novo CNVs**	**SNP 6.0**	**2.7 M**	**Omni**	**CytoSNP**	**CGX-12** ^**a**^	**All arrays**
**Total**	139	150	35	8	56	388
Gains	108	121	7	4	25	265
Losses	31	29	28	4	31	123
Range per patient	0-17	0-20	0-4	0-2	0-7	10-30
Median per patient	7	6	2	0	3	16
Average per patient	6.6	7.1	1.7	0.4	2.7	18.5
**Benign** (% of total)	**36** (25.9%)	**1** (0.7%)	**14** (40.0%)	**1** (12.5%)	**51** (91.1%)	**103** (26.5%)
Gains	22	0	3	1	23	49
Losses	14	1	11	0	28	54
Range per patient	0-3	0-1	0-4	0-1	0-7	2-13
Median per patient	2	0	0	0	3	5
Average per patient	1.7	0.0	0.7	0.05	2.4	4.9
Nb of patients (%)	17 (81.0%)	1 (4.8%)	7 (33.3%)	1 (4.8%)	20 (95.2%)	21 (100.0%)
**Unclear** (% of total)	**89** (64.0%)	**139** (92.7%)	**10** (28.6%)	**3** (37.5%)	**1** (1.8%)	**242** (62.4%)
Gains	75	115	1	2	1	194
Losses	14	24	9	1	0	48
Range per patient	0-15	0-19	0-2	0-1	0-1	5-24
Median per patient	4	6	0	0	0	9
Average per patient	4.2	6.6	0.5	0.1	0.05	11.5
Nb of patients (%)	20 (95.2%)	19 (90.5%)	7 (33.3%)	3 (14.3%)	1 (4.8%)	21 (100.0%)
**Pathogenic** (% of total)	**6** (4.3%)	**7** (4.7%)	**5** (14.3%)	**4** (50.0%)	**4** (7.1%)	**26** (6.7%)
Gains	3	4	2	1	1	11
Losses	3	3	3	3	3	15
Range per patient	0-4	1-2	0-3	0-2	0-2	1-13
Median per patient	0.5	1	0.5	0.5	0.5	3
Average per patient	1.0	1.2	0.8	0.7	0.7	4.3
Nb of patients (%)	3 (14.3%)	6 (28.6%)	3 (14.3%)	3 (14.3%)	3 (14.3%)	6 (28.6%)
**False positive (% of total)**	**8** (5.8%)	**3** (2.0%)	**6** (17.1%)	**0** (0.0%)	**0** (0.0%)	**17** (4.4%)
Gains	8	2	1	0	0	11
Losses	0	1	5	0	0	6
Range per patient	0-2	0-1	0-2	0	0	1-4
Median per patient	1	0	0	0	0	1
Average per patient	0.8	0.3	0.6	0.0	0.0	1.7
Nb of patients (%)	6 (28.6%)	3 (14.3%)	4 (19.0%)	0 (0.0%)	0 (0.0%)	10 (47.6%)

Nb: number. Bold: CNV category breakdown.

^a^De novo or inheritance unknown (parents not systematically tested).

**Figure 1:**
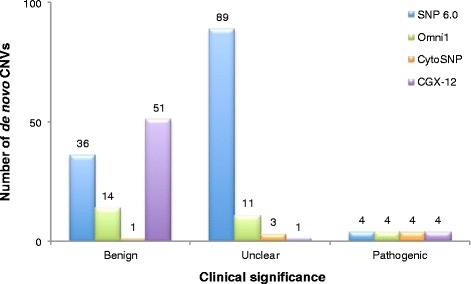
**Number of** ***de novo*** **CNVs detected by each array for all 21 patients, categorized according to clinical significance.**

**Figure 2:**
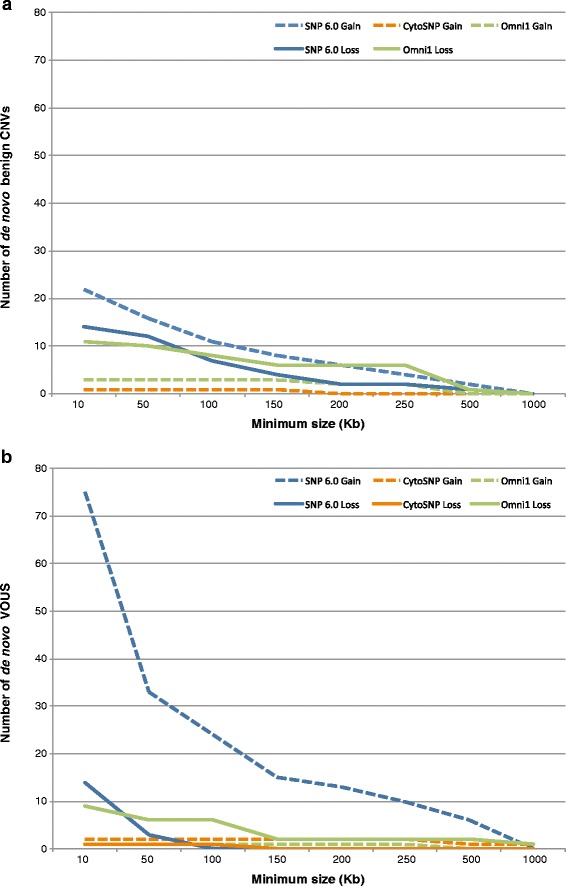
**Number of** ***de novo*** **CNVs detected by each array, as a function of minimum size. (a)** Benign CNVs. **(b)** VOUS.

There were some concerns about the accuracy of the results obtained with the 2.7 M platform version that we tested. Indeed, an extremely high proportion (139/150, 92.7%) of *de novo* CNV calls were VOUS, which was unexpected even considering the high density of this array. In addition, only one of the 150 CNV calls was benign, despite extensive coverage of polymorphic regions and segmental duplications, and only 24 of the 216 CNVs detected (inherited and *de novo*) were confirmed on another array. Therefore, we suspect a design or technical defect affecting CNV detection was responsible for these results. In order to have a meaningful comparison and for clarity purposes, we only report the results obtained with the other three arrays in the following section and in Figures 1 and 2. Information regarding *de novo* CNVs detected by 2.7 M is included in all the other sections and in all tables.

SNP 6.0 detected the highest number of benign CNVs with 36 (Figure 1), for an average of 1.7 per patient, compared to 0.7 for Omni1 and 0.05 for CytoSNP (Table 4). If we consider the total number of *de novo* CNVs detected, Omni1 had the highest proportion of benign CNVs (40.0% (14/35) vs 25.9% (36/139) for SNP 6.0 and 12.5% (1/8) for CytoSNP). In comparison, 91.1% of CNVs detected on CGX-12 were benign (51/56), for an average of 2.4 per patient. But this was expected since parents are not systematically tested on this array, thus a high proportion of these CNVs were probably in fact inherited. SNP 6.0 also had the highest number of VOUS with 89 (vs 10 for Omni1 and 3 for CytoSNP), for an average of 4.2 per patient (vs 0.5 for Omni1 and 0.1 for CytoSNP). Correspondingly, the highest proportion of VOUS was found by SNP 6.0, with 64.0% (89/139), compared to 28.6% (10/35) for Omni1 and 37.5% (3/8) for CytoSNP. In comparison, CGX-12 detected only one VOUS out of 56 “*de novo*” CNVs (1.8%), for an average of 0.05 per patient. Most of the VOUS detected by SNP 6.0 were gains (75 gains vs 14 losses), while benign CNVs were more equally distributed (22 gains vs 14 losses). Omni1 showed the opposite trend, both for VOUS (1 gain vs 9 losses) and benign CNVs (3 gains vs 11 losses). Interestingly, only four of the 89 VOUS detected by SNP 6.0 were also detected on at least another array (Additional file 4: Table S4), while a higher proportion of VOUS detected on CytoSNP and Omni1 were confirmed (2/3 and 4/10, respectively). In contrast, the confirmation rate of benign CNVs detected on SNP 6.0 was similar to the rate on Omni1 (7/36 confirmed for SNP 6.0, and 3/14 for Omni1), while it was 100% for CytoSNP (1/1). In addition, while size did not seem to impact the distribution of gains and losses for the benign CNVs (Figure 2a), the bias towards gains for the VOUS detected on the SNP 6.0 array appeared to be inversely correlated with the size of the CNV calls (Figure 2b). In summary, the SNP 6.0 array detected a higher proportion of gains in the VOUS category, most of which were of small size, whereas there was no such bias for the benign CNVs. The CytoSNP array did not show a bias in either category, while the Omni1 array identified more losses than gains (both in the benign and VOUS categories). In addition, the confirmation rate of VOUS was lower for the SNP 6.0 array than for the Illumina arrays, while there was no difference in the confirmation rate of benign CNVs.

Finally, SNP 6.0 called six pathogenic CNVs (6/139, 4.3%) while Omni1 called five (5/35, 4.7%) and CytoSNP called four (4/8, 50%), but these all correspond to the same four pathogenic alterations that were fragmented into smaller segments by SNP 6.0 and Omni1, thus none of the tested arrays identified a pathogenic alteration that was undetected by the other arrays. Therefore, these three arrays found a pathogenic CNV in 14.3% of patients (3/21, one patient had two CNVs), which corresponds to the results obtained on the CGX-12 array (details in the next section). The main differences resides in the total number of CNV calls that had to be interpreted for each patient (Table 4), which ranged from an average of 1.7 per patient (range 0–4) for Omni1 and 0.4 (range 0–2) for CytoSNP, to 6.6 (range 0–17) for SNP 6.0, and the proportion of patients for whom we identified at least one VOUS, which was 14.3% for CytoSNP (3/21), 33.3% for Omni1 (7/21) and 95.2% for SNP 6.0 (20/21). In comparison, for CGX-12, the average number of CNV calls to interpret for each patient was 2.7 (range 0–7), and the proportion of patients with a VOUS was 4.8% (1/21). In summary, all arrays detected the pathogenic CNVs, but the overall number of CNV calls to interpret for each patient was higher for SNP 6.0.

### Clinical significance of confirmed *de novo* CNVs

Of the 332 *de novo* CNVs detected on the four tested arrays, 47 were confirmed (detected on at least two arrays), and they represented 20 unique alterations (Additional file 4: Table S4). Many of these showed variations in the breakpoints delineated by each array. Four of these alterations, in three patients, were classified as pathogenic, ten were classified as benign, and six were classified as VOUS. All pathogenic CNVs and two of the benign CNVs (patients 26.9 and 50.17) were also detected on CGX-12. One of the VOUS was deemed benign on CGX-12 (patient 32.11), because it was called as a heterozygous loss in a polymorphic region, but Omni1 and CytoSNP called a homozygous deletion (CN = 0), hence the uncertain clinical significance interpretation. Only five CNVs were called on all four tested arrays: the four pathogenic CNVs, and one VOUS (patient 3.2, Additional file 5: Figure S1). This copy number gain could be seen retrospectively on CGX-12, but was not called by the algorithm.

In patient 14.5, CGX-12 found a 6.27 Mb gain of 6p25.3p25.1 and a 3.9 Mb loss of 7q36.3, resulting from an unbalanced 6;7 translocation (Additional file 6: Figure S2). All four tested arrays successfully detected these alterations, although 2.7 M and Omni1 fragmented the chromosome 6 gain into two smaller segments, and SNP 6.0 fragmented it into three segments.

In patient 41.14, CGX-12 identified a 2.8 Mb deletion of 12q13.12q13.13 that was also detected by all tested arrays as a single segment, except for 2.7 M, which fragmented this loss into 4 segments (Additional file 7: Figure S3).

In patient 44.15, CGX-12 detected a 2.5 Mb deletion in the DiGeorge/Velocardiofacial syndrome region (OMIM 188400/192430) on chromosome 22 (Additional file 8: Figure S4). SNP 6.0 and Cyto SNP both detected the deletion, while Omni1 fragmented this loss into two segments (1.43 Mb and 756 kb). The gap between these two segments is rich in segmental duplications and is not well covered by the other arrays. 2.7 M only detected a 877 kb loss.

### UPD

Three patients (1.1, 10.4 and 11.4), in two families, had LCSHs larger than 10 Mb. There were differences between arrays concerning the detection and the size of these LCSHs (Additional file 9: Table S5). All large LSCHs were successfully detected by SNP 6.0 (except one that was just below threshold at 9999.286 kb) and CytoSNP, with a few differences in size. However, Omni1 detected only 9 of the 21 large LSCHs, and 2.7 M detected 18 of them. The remaining LCSHs were fragmented and thus below the 10 Mb threshold, and even below the 5 Mb threshold in some cases on the 2.7 M array. All three patients had multiple large LCSHs on more than one chromosome, suggesting parental consanguinity. Since CytoSNP detected all LCSHs larger than 10 Mb, we report these results in the rest of this section. Patient 1.1 had nine LCSHs larger than 10 Mb, for a total of 150.239 Mb, and an estimated percentage of homozygosity of 5.2% (Additional file 10: Table S6). This was consistent with third degree consanguinity (coefficient of inbreeding (F): 0.0625). Patient 10.4 and 11.4, who are siblings, had six LCSHs each, for a total of 159.919 Mb and 168.894 Mb, respectively. The percentage of homozygosity was respectively 5.6% and 5.8%, which was also consistent with third degree consanguinity. Parental consanguinity was confirmed with family history obtained from the parents.

### Informative LCSHs

The four tested arrays detected a total of 112 LCSHs larger than 5 Mb, representing 28 unique homozygous regions in five patients (Additional file 9: Table S5). The 2.7 M array detected the highest number with 31, of which 29 were confirmed on other arrays, but with important differences in size. The two remaining LCSHs were in fact detected on other arrays, but with a size below the 5 Mb threshold. The SNP 6.0 array detected 26 LCSHs larger than 5 Mb in five patients, all of which were detected by CytoSNP and 2.7 M, except two: a 5.2 Mb LCSH on chromosome X (female patient 59.20) in a region sparsely covered by CytoSNP, and a 15.8 Mb LCSH on chromosome 16 (patient 1.1), which was fragmented into four segments smaller than the 5 Mb threshold by 2.7 M. CytoSNP detected 25 LCSHs larger than 5 Mb, all of which were confirmed at least on the SNP 6.0 array. Finally, Omni1 detected 30 LCSHs in three patients (1.1, 10.4 and 11.4), many of which were fragments of the larger LCSHs found on the other arrays. Omni1 did not detect the LCSH in patient 59.20, despite adequate coverage, and detected the LCSH in patient 26.9, but fragmented it in two smaller segments that were below the 5 Mb threshold. Of the 25 confirmed LCSHs, 24 were found in patients born from consanguineous parents (patients 1.1, 10.4 and 11.4) and one was found in patient 26.9 (no consanguinity). Four of these LCSHs (in three families) were considered informative (Table 5), because they overlap a gene associated with a recessive condition compatible with the patient’s phenotype. Three of them were confirmed to harbour a pathogenic homozygous mutation.

**Table 5:** **Informative LCSHs larger than 5 Mb**

**Patient**	**LCSH**	**Array**	**Chr**	**Chr band(s)**	**Coordinates**		**Size (kb)**	**Gene(s) of interest & disorder**	**Confirmed?**
					**Start**	**End**			
1.1	7	SNP 6.0	8	q21.11 q23.1	74259208	106471063	32 212	*VPS13B*, Cohen syndrome	No. Known polymorphism identified by exon sequencing (c.T9492C, Homozygous C).
		2.7 M		q21.11 q22.3	74144714	103958979	29 814		
		Omni1		q21.11 q23.1	74275315	106474543	32 199		
		CytoSNP		q21.11 q23.1	74256250	106477669	32 221		
10.4	16	SNP 6.0	11	q14.2 q23.1	86714942	118761985	32 047	*DPAGT1*, Congenital disorder of glycosylation, type 1j (CDG1J), *ALG9*, Congenital disorder of glycosylation, type 1 l (CDG1L)	Yes. Homozygous c.A322G mutation in exon 3 of *DPAGT1* identified by exome sequencing.
		2.7 M		q14.2 q23.1	86640025	112683203	26 043		
		CytoSNP		q14.2 q23.3	86714943	118727916	32 013		
11.4	24	SNP 6.0	11	q14.1 q24.2	81709865	124226213	42 516	*DPAGT1*, Congenital disorder of glycosylation, type 1j (CDG1J), *ALG9*, Congenital disorder of glycosylation, type 1 l (CDG1L)	Yes. Homozygous c.A322G mutation in exon 3 of *DPAGT1* identified by exome sequencing.
		2.7 M		q14.1 q23.3	81631409	116681560	35 050		
				q23.3 q24.2	118899401	124319838	5 420		
		Omni1		q21 q22.3	93339399	103743297	10 404		
				q22.3 q24.2	106756061	124224315	17 468		
		CytoSNP		q14.1 q24.2	81730470	124222740	42 492		
26.9	27	SNP 6.0	2	p21	41858417	46908485	5 050	*LRPPRC*, Leigh syndrome, French-Canadian type (LSFC)	Yes. Homozygous c.C1119T mutation in exon 9, corresponding to the known major p.A354V present in 97.6% of affected LSFC.
		2.7 M		p21	41794186	46981017	5 187		
		CytoSNP		p21	41850116	46912030	5 062		

Chr: chromosome.

Patient 1.1 had a 32.2 Mb LCSH in 8q21.11q23.1 (Additional file 11: Figure S5), which contains *VPS13B* (OMIM 616817). Patients with Cohen syndrome (OMIM 216550) are homozygous or compound heterozygous for mutations in this gene. Cohen syndrome was amongst the suspected diagnoses for this patient, but exon sequencing only identified a synonymous mutation in exon 54, which is a known polymorphism found in healthy individuals.

Patient 11.4 had a 42.5 Mb LCSH in 11q14.1q24.2, which completely overlaps with a 32.0 Mb LCSH in 11q14.2q23.3 found in his brother, patient 10.4 (Additional file 12: Figure S6). The overlapping region contains *DPAGT1* (OMIM 191350) and *ALG9* (OMIM 606941). Homozygous or compound heterozygous mutations in either of these genes cause Congenital disorder of glycosylation, type 1j (OMIM 608093) and type1l (OMIM 608776), respectively. Both patients have DD, hypotonia, and abnormal transferrin glycosylation. Exome sequencing confirmed a homozygous A322G mutation in exon 3 of *DPAGT1* in both patients. This mutation has not been reported before, but segregates with disease in this family, and is predicted to be “deleterious” and “possibly damaging” by two mutation effect prediction tools [34,35]. Therefore, this mutation is very likely to be pathogenic.

Patient 26.9 had a 5 Mb LCSH in 2p21 (Additional file 13: Figure S7) that overlaps with *LRPPRC* (OMIM 607544). Homozygous and compound heterozygous mutations in this gene have been identified in patients affected with Leigh syndrome, French-Canadian type (OMIM 220111). This patient has DD, ataxia, lactic acidosis, ptosis, and obesity. He was confirmed to be homozygous for the A354V mutation in exon 9 of *LRPPRC*, which is found in most patients with French-Canadian type Leigh syndrome.

### Interpretation software

Since establishing clinical significance of detected CNVs can be challenging, and the software used to visualize results influences interpretation efficiency, we also compared the cytogenetics software provided by the vendors in order to have a complete assessment of each platform’s performance. ChAS (Affymetrix) and KaryoStudio (Illumina) allow array results analysis, interpretation and reporting by visualizing data in a chromosome and genome view. Both softwares offer various features that are detailed in Additional file 14: Table S7. In ChAS, every item visualized in “Detailed view” is clickable and links to either the detailed information in tables (“Called segments”) or the external database entry (RefSeq gene, OMIM, DGV), which made it easier to follow the same interpretation workflow for each CNV call and avoid missing important information. In KaryoStudio, the lack of links between the graphical view, the external databases (except RefSeq) and the information in tables made interpretation approximate, time-consuming and more prone to errors. In addition, although KaryoStudio offers the possibility of simultaneously visualizing results for a trio (patient and parents), called segments appear only for the patient, and have to be compared with the LogR or B allele frequency (BAF) to establish inheritance. For some CNV calls, the parent’s signal was clear, but for others it was not, and in these cases, there was no way to visually confirm if the same CNV was called in the parent. The only option was to search the “Detected Regions” table and compare genomic coordinates. One useful feature available in KaryoStudio is the ability to save interpretation information in a “Comments” column. This was not possible in ChAS, where there is an “Interpretation” column that can be exported with a table report, but the information entered cannot be saved within ChAS. Finally, both softwares offer a report feature that exports a graphical view and the details of the abnormal region. KaryoStudio exports results for all patients in the same batch with a simple click, while every abnormal region has to be exported individually in ChAS. However, with ChAS, the user can choose exactly which tracks to export, and how it is presented (individual or trio, zoomed or not). In summary, although KaryoStudio’s interface is simpler, CNV interpretation was easier in ChAS.

## Discussion

In the present study, we evaluated the diagnostic performance, in a clinical context, of four high-resolution SNP arrays by testing 21 patients affected with ID/DD and comparing the results obtained on each one. A diagnosis was successfully made in six patients (28.6%, 6/21) from 5 families (25.0%, 5/20): four pathogenic CNVs (including one unbalanced translocation) and three LCSHs harbouring a pathogenic homozygous mutation were detected. However, our results also highlight the challenges associated with the use of high-resolution arrays at low detection thresholds, and identify some of the issues specific to each array. Specifically, all arrays successfully picked up the pathogenic abnormalities, but with enough variation in size and breakpoints to have an impact on the quality and accuracy of the final reported results. Another major difference between arrays was observed in the overall number of CNVs detected by each platform, which has a significant impact on workload. Nonetheless, our results show that the tested SNP arrays are effective at identifying large CNVs, and that there is an added value in detecting and analysing LCSHs. Indeed, as a result of the homozygous mutations uncovered, the overall diagnostic yield was higher (28.6%, 6/21) than the yield we would have obtained with the platform we currently use (14.3%, 3/21), as well as the yield reported in previous studies using high resolution SNP arrays but restricted to CNV detection, which ranges from 4.4% to 16% [36-40].

### CNVs

Recent studies performed with high-resolution arrays [19,20,27] show the usefulness of these arrays by focusing on the pathogenic CNVs identified without taking into account the overall number of CNVs detected for each patient, therefore ignoring a critical aspect in a clinical setting: the time allocated to CNV interpretation and the challenges associated with result reporting. With standard thresholds, it was clear that both Affymetrix arrays detected too many CNVs for each patient (average of 24.0 and 17.5 per patient, for SNP 6.0 and 2.7 M respectively, see Additional file 1: Table S1) to be used in a clinical laboratory. Indeed, we want to continue detecting small (<200 kb) pathogenic alterations — the whole-genome array we currently use can detect pathogenic CNVs as small as 7 kb — while keeping the total number of CNVs to interpret for each patient to a manageable level. Filtering out benign variants and using different thresholds according to gene content proved to efficiently reduce the overall number of CNVs detected per patient (see Additional file 2: Table S2) to an average of 11.9 (SNP 6.0) and 10.3 (2.7 M), which was comparable to what the Omni1 array detected (average 8.8), but still much higher than CytoSNP (average 1.2) and CGX-12 (average 2.8). Considering that the first three arrays have a much higher density of markers than CytoSNP and CGX-12, this higher number of detected CNVs was expected, and could nevertheless represent an advantage if it results in the detection of additional small pathogenic alterations.

It is noteworthy that although 80% of the CNVs detected on the Affymetrix arrays were gains (see Additional file 2: Table S2), a much lower proportion of gains (compared to losses) were confirmed (see Additional file 3: Table 3). In contrast, there was no difference between gains and losses confirmation rates for the Illumina and the NimbleGen arrays. For the SNP 6.0 array, this bias towards gains was more important as the size of the alterations decreased, but only for VOUS (see Figure 2). In addition, no gains smaller than 150 kb on the Affymetrix arrays were confirmed, but larger gains and small losses were (see Additional file 4: Table S4). This bias was seen in all tested patients, which suggests that there was a technical or design flaw affecting small gains (either false positives on the Affymetrix arrays, or false negatives on the Illumina arrays) that would limit the validity of the results obtained with the problematic array(s). Assessing the cause(s) of these issues would require an in-depth analysis of the data quality metrics of each platform, and may represent a limitation of this study, which was focused on the clinical application of the platforms as provided by the manufacturers.

In addition to the overall number of CNVs detected by the Affymetrix arrays being an issue, the proportion of *de novo* VOUS was also a concern, especially considering the fact that most of them were not confirmed on another array. Although the problem seemed less important on SNP 6.0 than on 2.7 M (92.7% of the *de novo* CNVs detected by 2.7 M were VOUS, of which only two were confirmed), the SNP 6.0 array still detected a total of 89 VOUS (64.0%; 89/139), in 20 of the 21 patients, and only four of them were confirmed. This extremely low confirmation rate was not seen with the benign CNVs, for which Affymetrix had rates comparable to Illumina. Since the majority of these VOUS were gains, it supports a technical issue with the detection of copy number gains on the Affymetrix arrays. Once again, the specific cause(s) for this low confirmation rate is difficult to assess without performing a thorough analysis of the quality metrics. In contrast, CytoSNP identified a VOUS in only three patients and Omni1 found ten VOUS in seven patients. Moreover, a higher proportion of them were confirmed on another array (2/3 and 4/10). Our results are consistent with the expectation that increased array resolution would lead to an increase in the overall number of CNVs detected, as well as VOUS. These proportions are more manageable on the Illumina arrays even if higher than CGX-12, which identified only one VOUS. This 45 kb gain was also seen on the SNP 6.0 and Omni1 arrays, but not called because it was below the size threshold (100 kb in regions without genes of known clinical significance. This last example confirms that using differential thresholds according to gene content is an effective approach to reduce the number of VOUS detected.

Even if SNP 6.0 and Omni1 detected a high proportion of benign CNVs, this was still lower than the results obtained on CGX-12. In addition, the proportion of confirmed benign CNVs was also low (see Additional file 4: Table S4), but contrary to the confirmation rate of VOUS, the rates were similar for Affymetrix and Illumina arrays. Although detecting a high number of benign CNVs added to the interpretation workload, this was less of an issue since the mere fact of being able to classify them as benign means their interpretation was fairly straightforward. The fact that parents were not systematically tested, and the use of a pool of six individuals as reference DNA probably explain why most CNVs identified on CGX-12 were classified as benign. The reference DNA used was also likely responsible for the low confirmation rate and the discrepancies observed in the copy number states called by different arrays for a same segment in some patients, particularly in regions prone to variations in normal individuals. For example, CGX-12 detected a 103 kb gain at 8p11.23 that was deemed benign in patient 38.13, while Omni1 identified an overlapping 154 kb inherited loss in the same patient (see Additional file 3: Table S3). There were numerous examples of this, and it affected all arrays. This illustrates the importance of referring to normal variants databases that are complete and contain information from various studies, as well as in-house created databases.

We identified a total of four pathogenic CNVs in three patients, and all tested arrays detected them. However, in some instances, there were important differences in the breakpoints called by each array (data not shown). The 2.7 M array was particularly problematic: it fragmented two of the four pathogenic CNVs, and called a third CNV less than half the size reported by all the other arrays. Omni1 also fragmented the pathogenic deletion identified in patient 44.15 into two smaller segments, separated by a 350 kb gap with a normal copy number state. However, Omni1 is the only one of the five arrays that has a dense coverage of the genomic region in that gap, which is rich in segmental duplications and normal variants. This type of fragmentation in highly polymorphic regions is likely more representative of the true copy number state, and although its interpretation should be fairly easy, it could complicate result reporting. For this patient, the larger CNV would still have been considered pathogenic, but the smaller deleted segment would have been assigned an uncertain clinical significance. The other variations in breakpoints and fragmentation were less important, and would not have had any significant impact in the clinical interpretation of the results.

The high number of CNVs detected for each patient on some arrays may have been acceptable if it had also resulted in an increase in diagnostic yield, by allowing the detection of small pathogenic alterations that could not be seen on lower density arrays. However, that was not the case in our study, nor was it in another study applying uniform thresholds for all patients [41]. This may be due to a small sample size, since two other studies found pathogenic CNVs smaller than 100 kb in a cohort of 6500 and 82 patients [20,27], respectively. However, these reports also suggest that decreasing detection thresholds is not sufficient to improve diagnostic yield. Indeed, the thresholds used in the first study [27] were not specifically defined, and they were possibly not the same for all tested patients since they were influenced by patients’ phenotype information. In the second study [20], a separate confirmation technique — in addition to various quality control parameters — was needed in order to reduce the false positive detection rate from 60% to 25%. This may be justifiable in a research setting, but we suspect the workload and the proportion of VOUS in these studies were tremendously important and would be difficult to sustain in a clinical setting. In addition, there should not be a need to confirm detected CNVs once an array has been properly validated [42,43], although doing so may be indicated to rule out mosaicism, or to visualize structural CNV location. These two studies and our results highlight the need to improve how we target potentially relevant genomic regions in order to increase the efficiency of high-resolution arrays, and use them to their full potential. Automating CNV interpretation with computational methods such as GECCO [44], and decreasing the proportion of VOUS with more complete variants databases could also eventually allow the detection and interpretation of a higher number of CNVs for each patient and the identification of more pathogenic CNVs.

### LCSHs & UPD

None of the patients tested in our study had UPD (data not shown), therefore it was not possible to directly assess the arrays capacity at detecting it. In addition, differentiating UPD from autozygosity in patients from consanguineous families would not be possible without performing trio analysis in another software. Nevertheless, with the method we applied, results from SNP 6.0 and CytoSNP were highly concordant and lead to no false positives. Therefore, it is reasonable to believe that their performance would have been adequate. In fact, two prospective studies successfully detected UPD using these arrays with thresholds similar to ours [31,45]. The performance of 2.7 M and Omni1 is more difficult to predict, since 2.7 M missed three large LCSHs and Omni1 detected less than half of LCSHs larger than > 10 Mb. Because it tended to fragment LCSHs, using a method that takes into account smaller LCSHs [46] could be more appropriate for Omni1, although doing so might increase the number of false positives, especially when patients come from a population with some degree of inbreeding. It is more plausible that higher LCSHs size thresholds, combined with individual chromosome size and LCSHs size average will be the best approach for UPD detection by SNP arrays. However, more prospective studies are needed to determine the optimal strategy for each array.

We were able to directly assess the capacity of each array to detect consanguinity, since parents were first cousins in two of the tested families. Three arrays successfully detected third degree consanguinity (SNP 6.0, 2.7 M and CytoSNP). The percentages of homozygosity obtained with Omni1 were suggestive of consanguinity in two patients, but too ambiguous to establish at which degree (2.5% and 2.3%, see Additional file 10: Table S6), and the percentage was too low in the third patient (0.5%). However, decreasing the minimum size of LCSH included in the calculation to 3 Mb would have improved the performance of all arrays, including Omni1. It has been suggested that a threshold of 10 Mb is sufficient to identify consanguinity and that lowering the minimum size of LCSHs included in the calculation has a negligible impact on the determination of percentage of homozygosity [32]. While a 10 Mb threshold is probably adequate for first-degree consanguinity, perhaps it is not for more distant consanguinity, and a lower threshold would be more appropriate in these cases.

Lastly, gene content of LCSHs larger than 5 Mb was also studied to uncover potential homozygous mutations causing autosomal recessive disorders. Once again, SNP 6.0 and CytoSNP showed the best concordance and detected all LCSHs — except one on chromosome X — while Omni1 and 2.7 M fragmented larger LCSHs into smaller segments, sometimes below the 5 Mb threshold. We followed up on four informative LCSHs — two in siblings, thus in the same genomic region — and successfully identified a pathogenic or likely pathogenic homozygous mutation in three of them. This resulted in a diagnosis for 14.3% of tested patients (3/21), and 66.6% of follow-ups (2/3). Previous studies reported a positive diagnosis in 0.08% of tested patients [45] — although in that report, the size threshold seems to have been variable — and in 10% of followed up LCSHs [32] (unknown thresholds). Our results clearly show the potential of SNP arrays in improving the diagnostic rate in patients with ID/DD, without unnecessarily burdening the workload, and compare advantageously to these reports. However, the expected yields might be overestimated because of our small sample size. The mutation suspected in the fourth LCSH, which was found in a patient with parental consanguinity, could not be confirmed. This LCSH was large and consequently, isolating a causative gene can be challenging.

Homozygosity mapping has been used for years in research, and more recently in clinical genetics. Combining it with molecular karyotype on SNP arrays undoubtedly facilitates its implementation and reduces overall costs [47]. The challenge resides, as with CNVs, in the workload associated with phenotype-genotype correlations, as illustrated by our failure to identify a causative mutation in one family. Because of the high amount of homozygosity present in all individuals, and even higher in consanguineous families, directing the analysis with a precisely defined phenotype seems unavoidable [27], and might even allow the use of very low threshold if the list of genes to verify for a specific phenotype is short. In addition, using an approach with fixed thresholds may not be optimal for LCSHs, since their number varies greatly from patient to patient, depending on their genetic background, and smaller LCSHs can also harbour homozygous mutations. It may indeed be more productive to adjust size thresholds for each patient in order to get a manageable number of LCSHs to further investigate. Automation can also play an important role in reducing the interpretation workload. As an example, Wierenga *et al.* recently developed an online tool that searches OMIM Clinical synopsis data to allow quick retrieval of the relevant genes within a genomic region, according to the patient’s phenotype [48]. More prospective clinical studies will be needed to establish a detailed algorithm and efficiently implement homozygosity mapping with SNP arrays in a clinical setting.

### Interpretation software

Interpretation and reporting of array results is the last but most important step in a clinical cytogenetics laboratory. It is a challenge in some cases, and requires easy and quick access to various databases containing information that is accurate and up-to-date. ChAS and KaryoStudio each have their pros and cons. In summary, we found that ChAS was much more complete and customizable, which allowed easy and thorough CNV interpretation, while saving and reporting results was much simpler in KaryoStudio. However, both softwares lacked features that are needed to make them functional as a stand-alone software in a clinical laboratory. The increase in resolution of the current arrays has resulted in a rise in the number of CNVs that are detected, and the interpretation process needs to be that much more efficient. Neither of the provided softwares would be adequate as they are, especially because they cannot be linked to an in-house laboratory database to keep track of past results. This is essential for many reasons, such as future reinterpretation of results as knowledge expands, but especially in the maintenance of each laboratory’s own database of normal variants, which vary depending on the population investigated or the platform used. There are a few softwares currently available that allow CNV (and/or LOH) visualization, interpretation, and database capabilities, such as Nexus Copy Number (BioDiscovery), CGHFusion (InfoQuant), and BENCH Lab CNV (Cartagenia) [49,50], and that are successfully used by various clinical laboratories [51-53]. While using them increases costs in the short term, arrays are constantly updated and improved, and laboratories needs may evolve. Therefore, this might represent the best approach in the long term, since it facilitates switching from one vendor to another, and allows using a different array depending on the clinical indication.

## Conclusions

High-resolution SNP arrays increase the diagnostic yield in patients with unexplained ID/MCA, compared to CGH microarrays, because of the information provided by the detection of LCSHs. However, the higher resolution does not increase the number of pathogenic CNVs detected by the arrays tested in our study, and there are differences in breakpoint accuracy, both for CNVs and LCSHs. In addition, the workload associated with the high number CNVs detected, the high proportion of VOUS identified (except for CytoSNP), and the inefficient visualization softwares provided by the vendors could make clinical implantation challenging. Therefore, *in silico* targeting of potentially pathogenic regions, interpretation automation, and the use of a commercial interpretation software and database may represent the best approaches that will allow the use of these arrays to their full potential in a clinical setting.

## Additional files

Additional file 1: Table S1. Number of CNVs detected by each tested array using standard thresholds. Additional file 2: Table S2. Number of CNVs detected by each tested array using optimized thresholds. Additional file 3: Table S3. List of CNVs confirmed on at least two arrays. Additional file 4: Table S4. List of de novo CNVs confirmed on at least two arrays. Additional file 5: Figure S1. VOUS detected in patient 3.2, as visualized in each software. 2p21 gain. (A) CytoSNP. (B) Omni1. (C) SNP 6.0 and 2.7 M. (D) CGX-12. Additional file 6: Figure S2. Pathogenic CNVs detected in patient 14.5, as visualized in each software. Der(7)t(6;7)(p25.1;q36.3). (A-D) 6p25.1pter gain. (A) CytoSNP. (B) Omni1. (C) SNP 6.0 and 2.7 M. (D) CGX-12. (E-H) 7q36.3qter loss. (E) CytoSNP. (F) Omni1. (G) SNP 6.0 and 2.7 M. (H) CGX-12. Additional file 7: Figure S3. Pathogenic CNV detected in patient 14.5, as visualized in each software. 12q13.12q13.13 loss. (A) CytoSNP. (B) Omni1. (C) SNP 6.0 and 2.7 M. (D) CGX-12. Additional file 8: Figure S4. Pathogenic CNV detected in patient 44.15, as visualized in each software. 22q11.21 loss. (A) CytoSNP. (B) Omni1. (C) SNP 6.0 and 2.7 M. (D) CGX-12. Additional file 9: Table S5. List of LCSHs larger than 5 Mb. Additional file 10: Table S6. Percentage of homozygosity calculated from copy-neutral LOHs. Additional file 11: Figure S5. Informative LCSH detected in patient 1.1, as visualized in each software. 32.2 Mb LCSH in 8q21.11q23.1, encompassing VPS13B. (A) CytoSNP. (B) Omni1. (C) SNP 6.0 and 2.7 M. Additional file 12: Figure S6. Informative LCSHs detected in patients 10.4 and 11.4, as visualized in each software. 32 Mb and 42 Mb LCSHs in 11q14.1q24.2, encompassing DPAGT1. (A-B) Patient 10.4. (A) CytoSNP. (B) Omni1. (C-D) Patient 11.4. (C) CytoSNP. (D) Omni1. (E) Patient 10.4 (upper part) and 11.4 (lower part), SNP 6.0 and 2.7 M. Additional file 13: Figure S7. Informative LCSH detected in patient 26.9, as visualized in each software. 5 Mb LCSH in 2p21, encompassing LRPPRC. (A) CytoSNP. (B) Omni1. (C) SNP 6.0 and 2.7 M. Additional file 14: Table S7. Software features and their impact on result interpretation.
