# Supplementary figures and images for: SNP arrays: comparing diagnostic yields for four platforms in children with developmental delay
Source: BMC Med Genomics. 2014 Dec 24;7:70. doi: 10.1186/s12920-014-0070-0 (PMC4299176; doi:10.1186/s12920-014-0070-0)

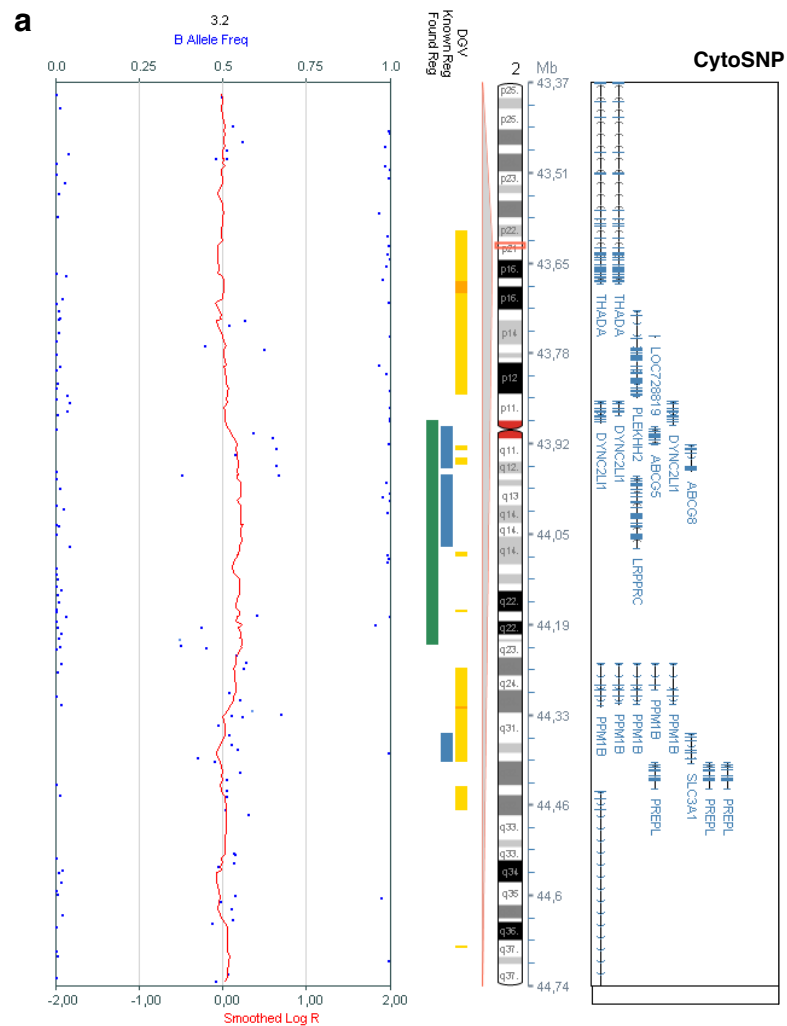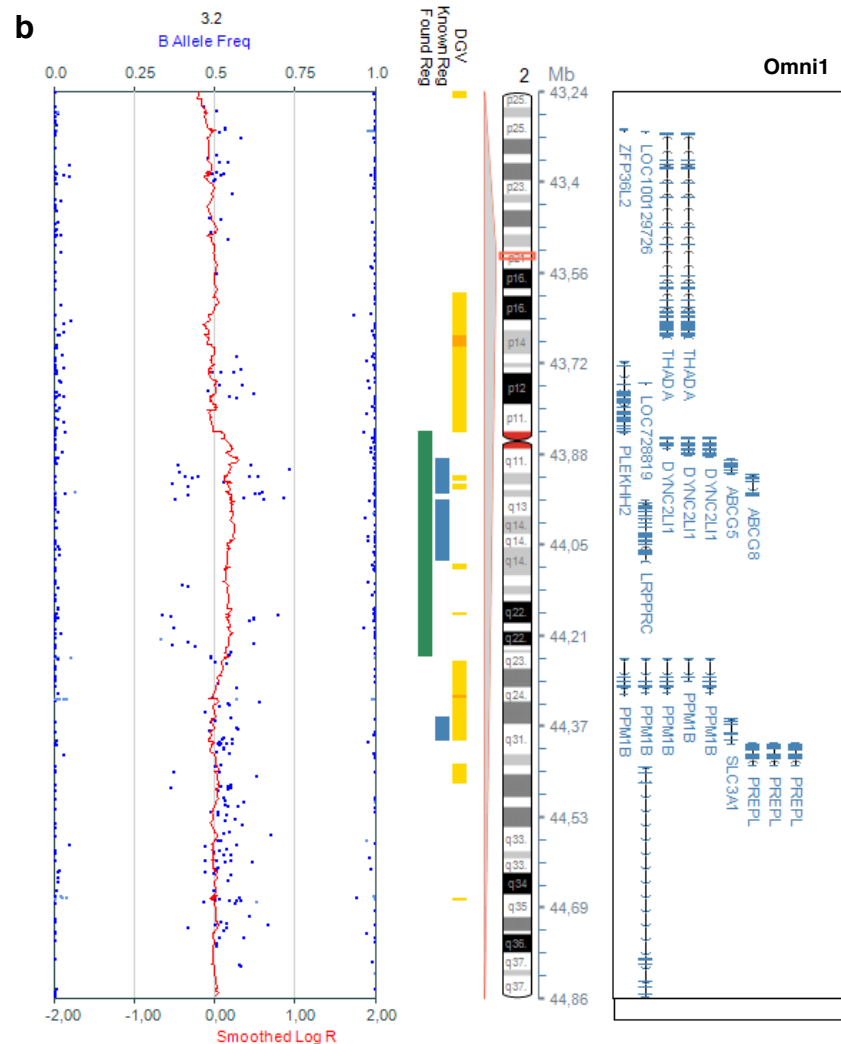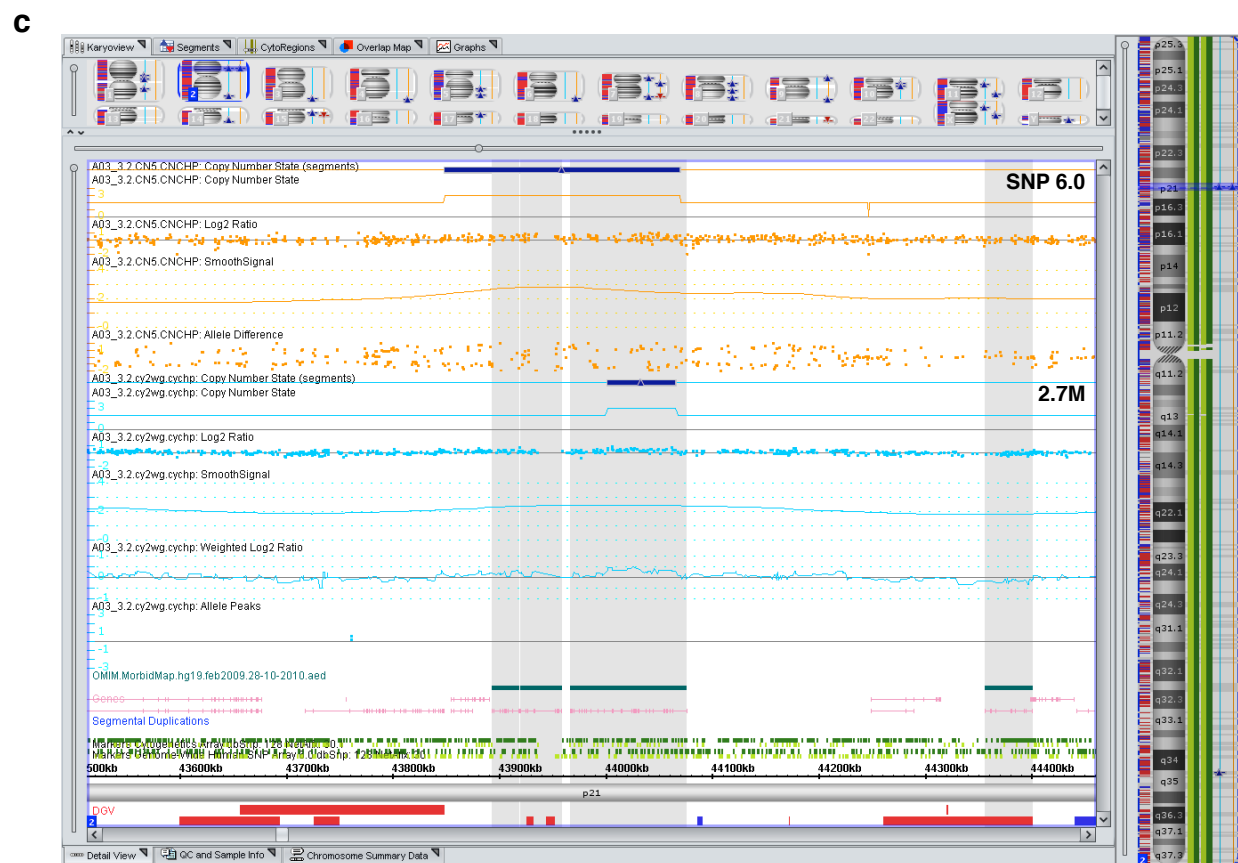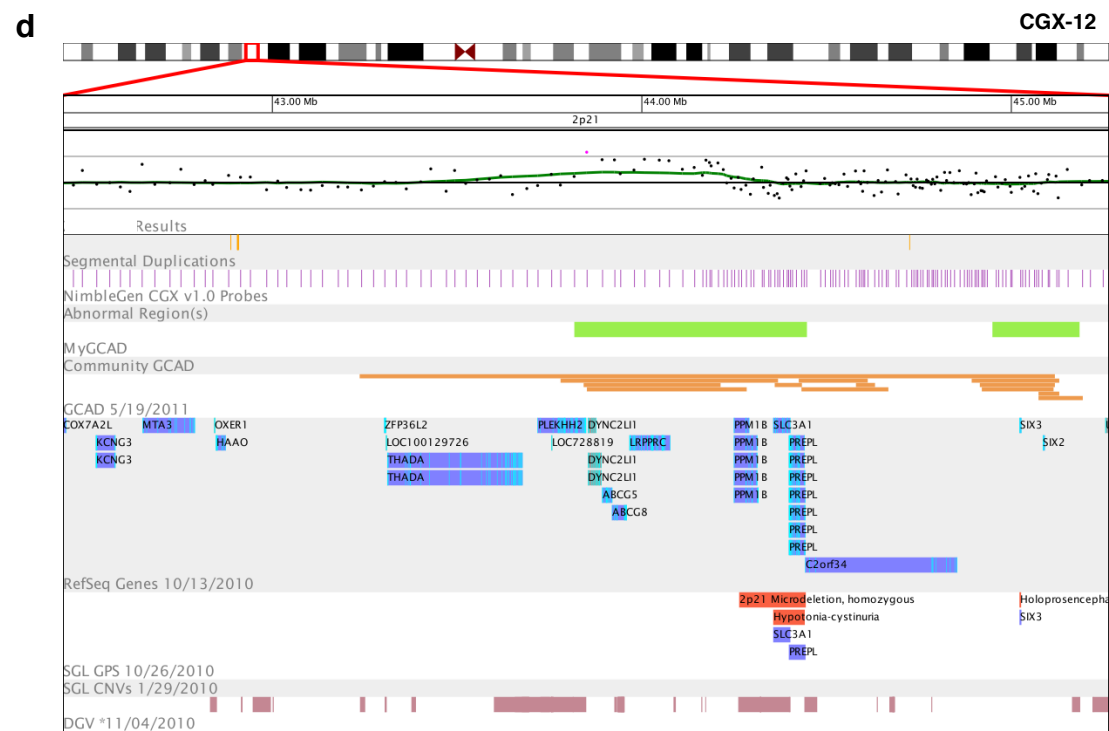

Supplement: Additional file 5: Figure S1. — VOUS detected in patient 3.2, as visualized in each software. 2p21 gain. (A) CytoSNP. (B) Omni1. (C) SNP 6.0 and 2.7 M. (D) CGX-12. [file 12920_2014_70_MOESM5_ESM.pdf]

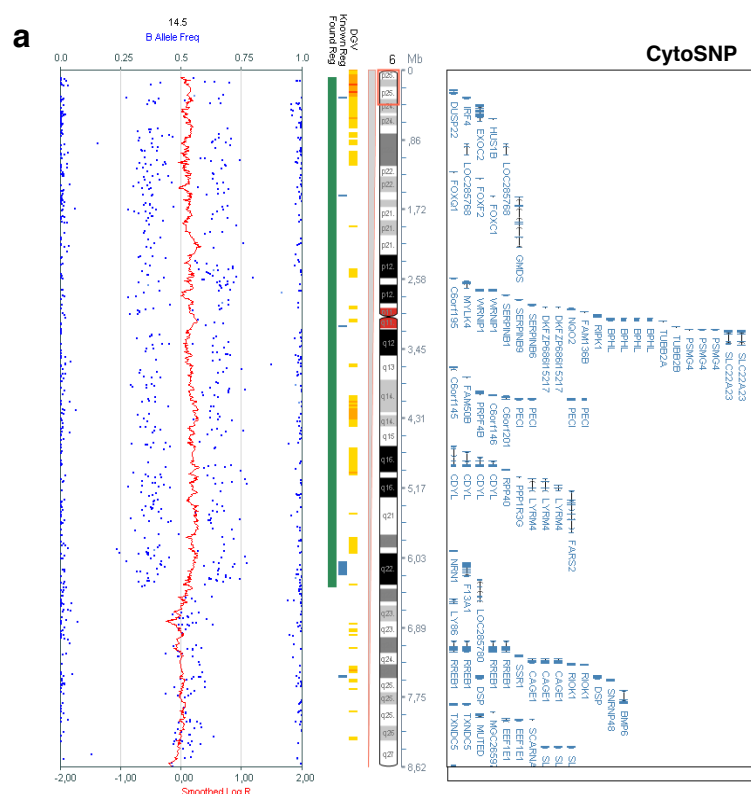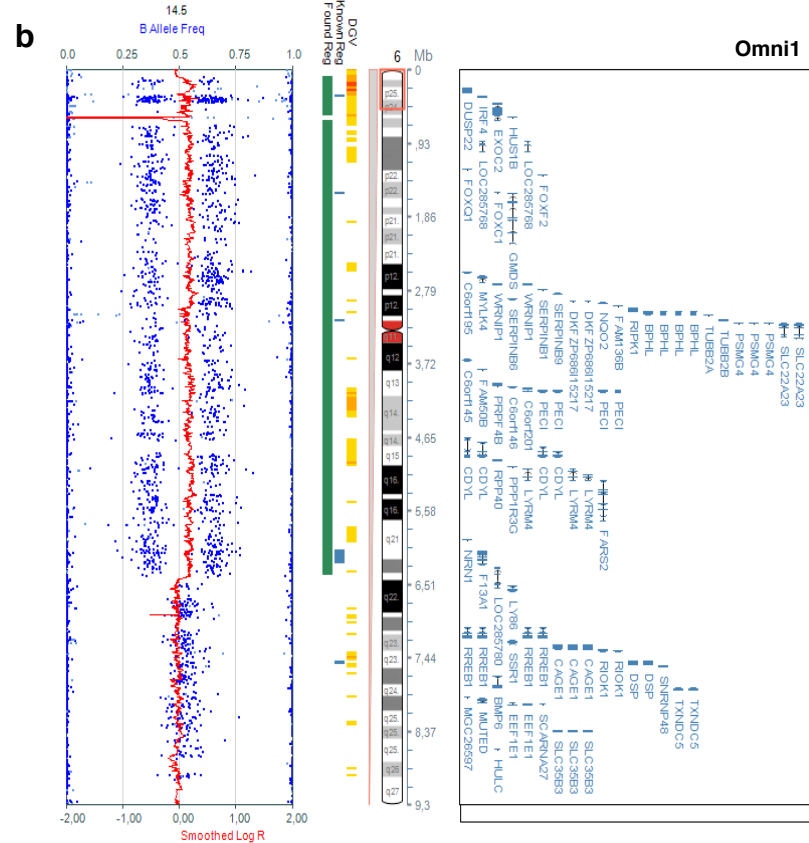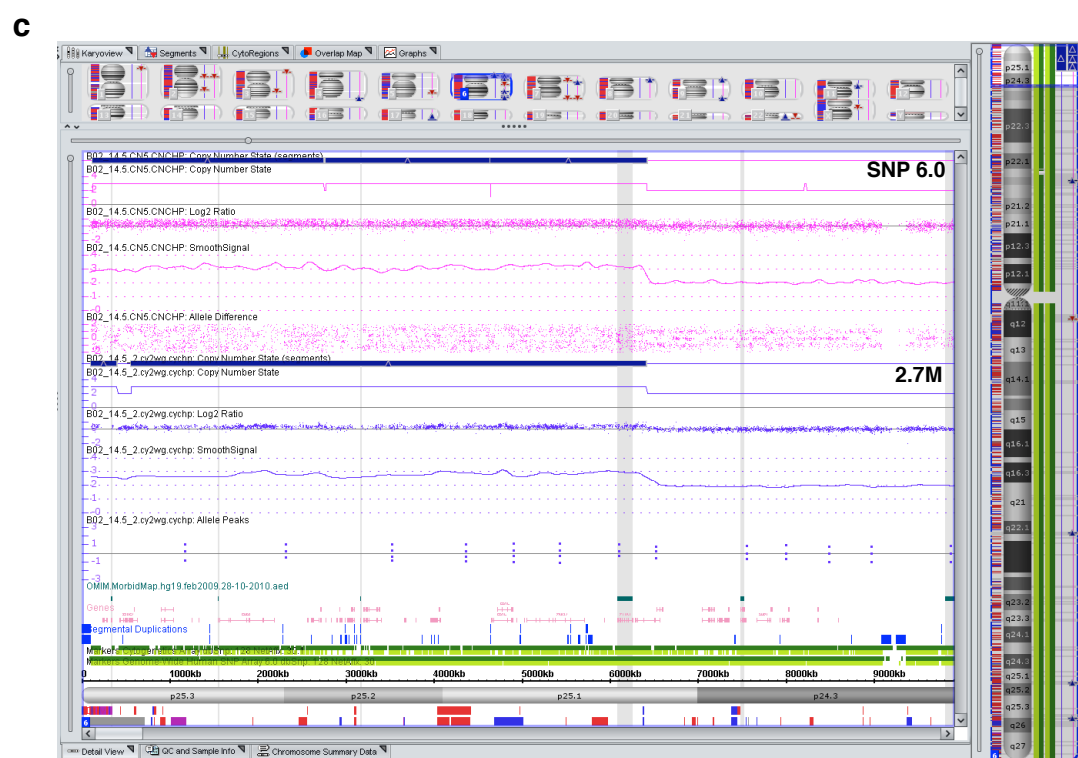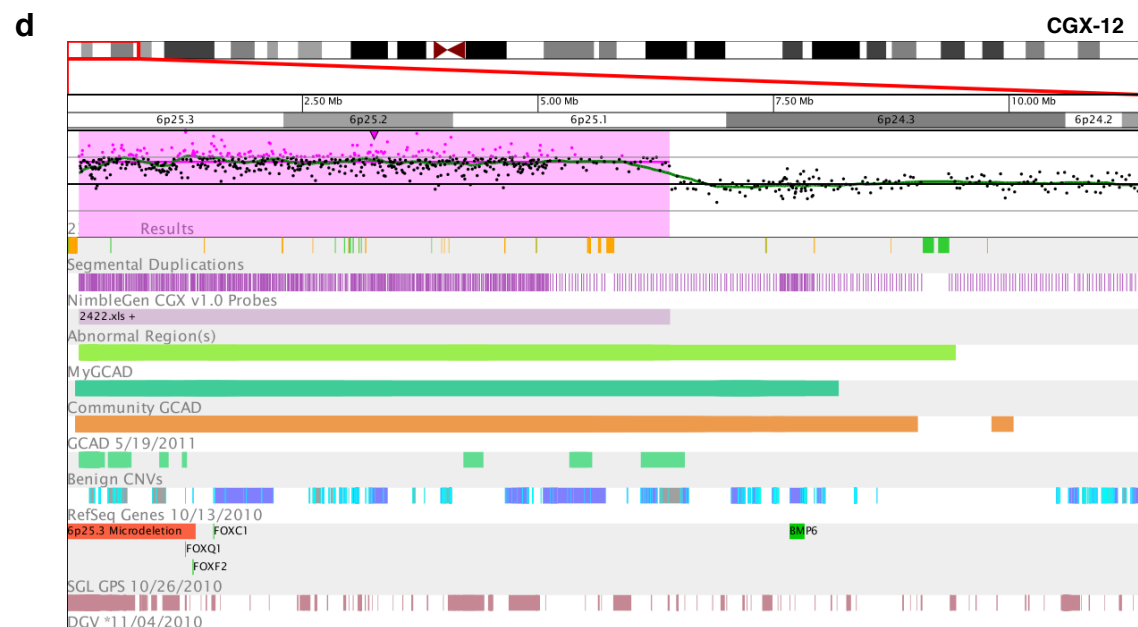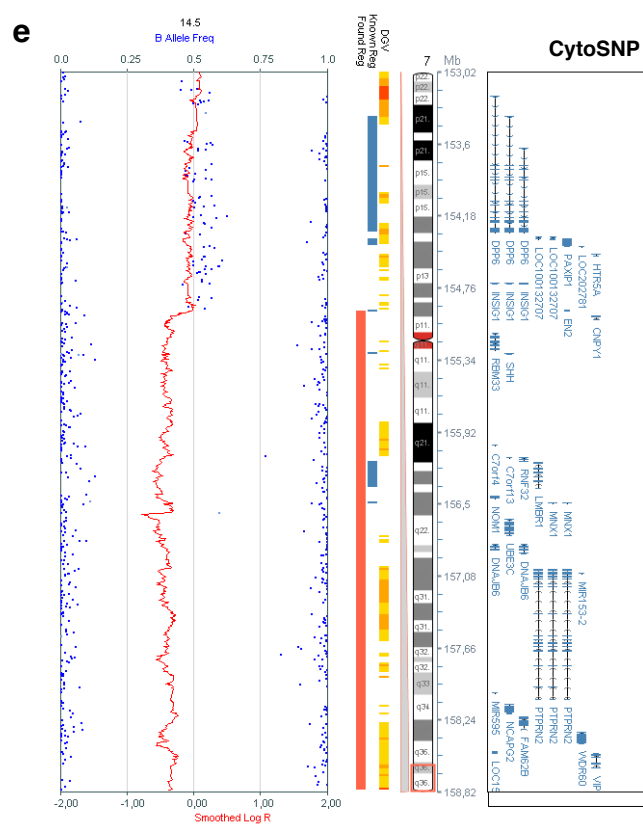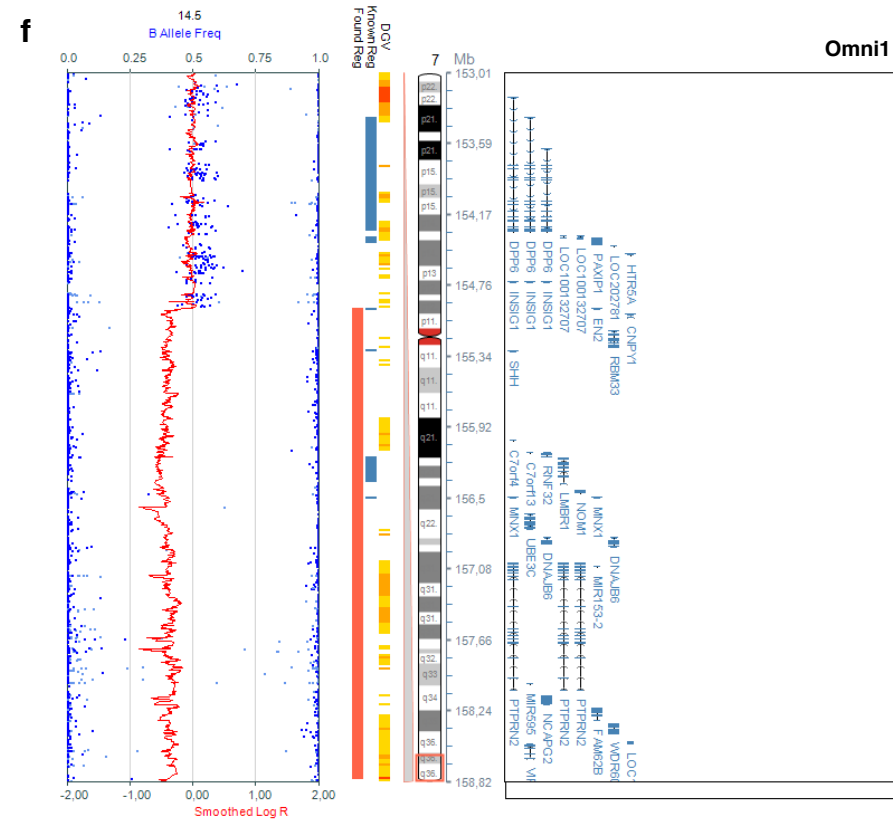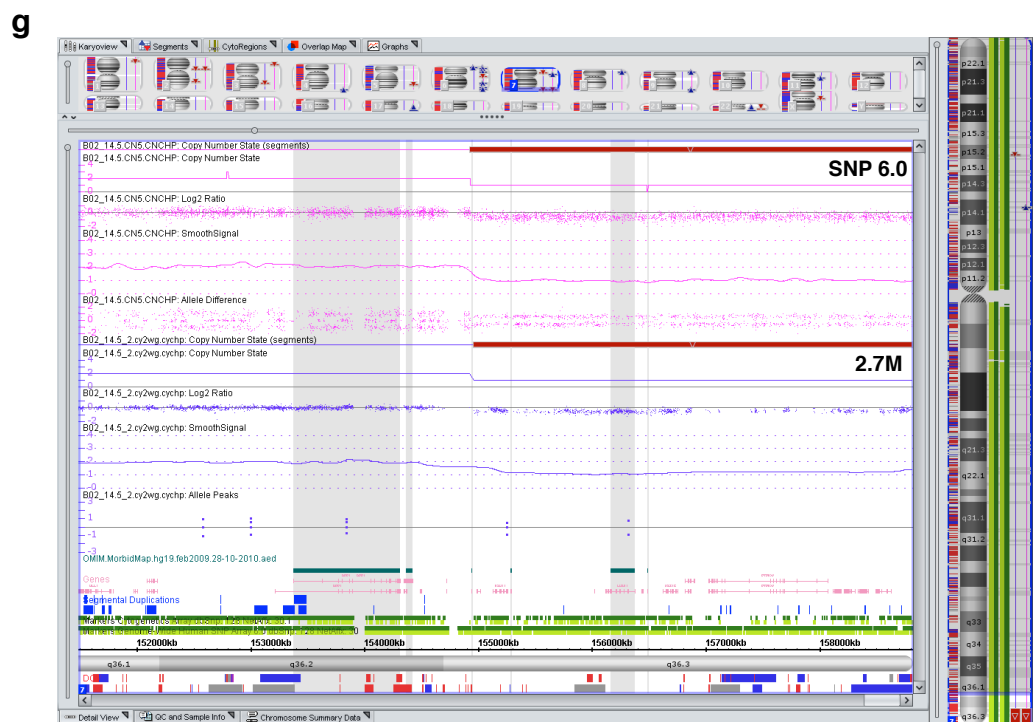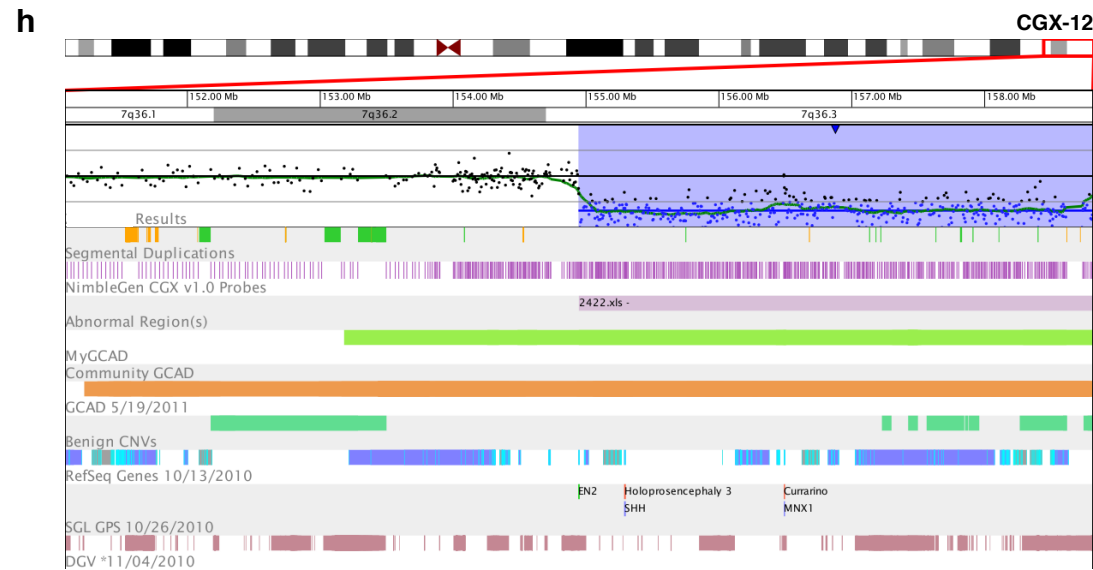

Supplement: Additional file 6: Figure S2. — Pathogenic CNVs detected in patient 14.5, as visualized in each software. Der(7)t(6;7)(p25.1;q36.3). (A-D) 6p25.1pter gain. (A) CytoSNP. (B) Omni1. (C) SNP 6.0 and 2.7 M. (D) CGX-12. (E-H) 7q36.3qter loss. (E) CytoSNP. (F) Omni1. (G) SNP 6.0 and 2.7 M. (H) CGX-12. [file 12920_2014_70_MOESM6_ESM.pdf]

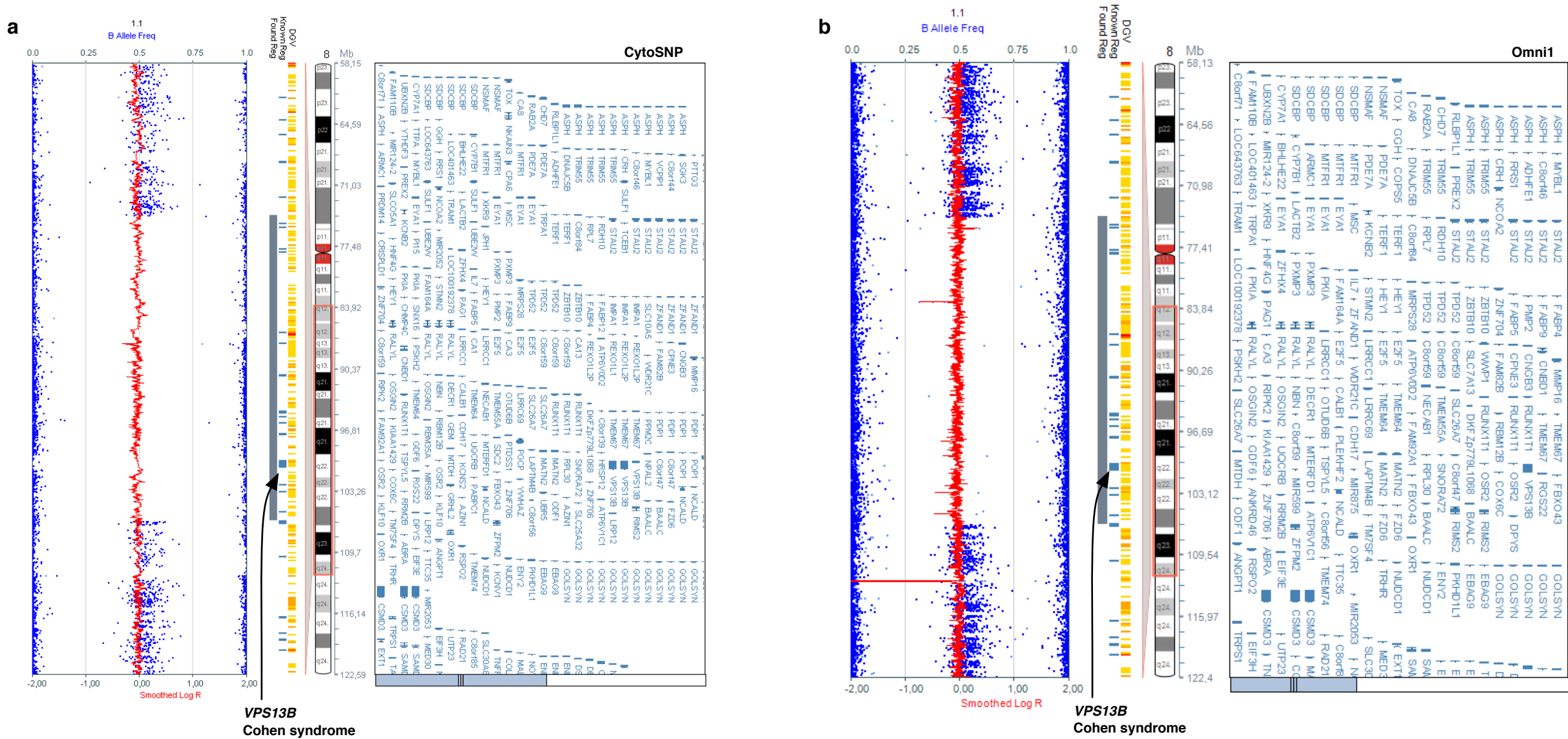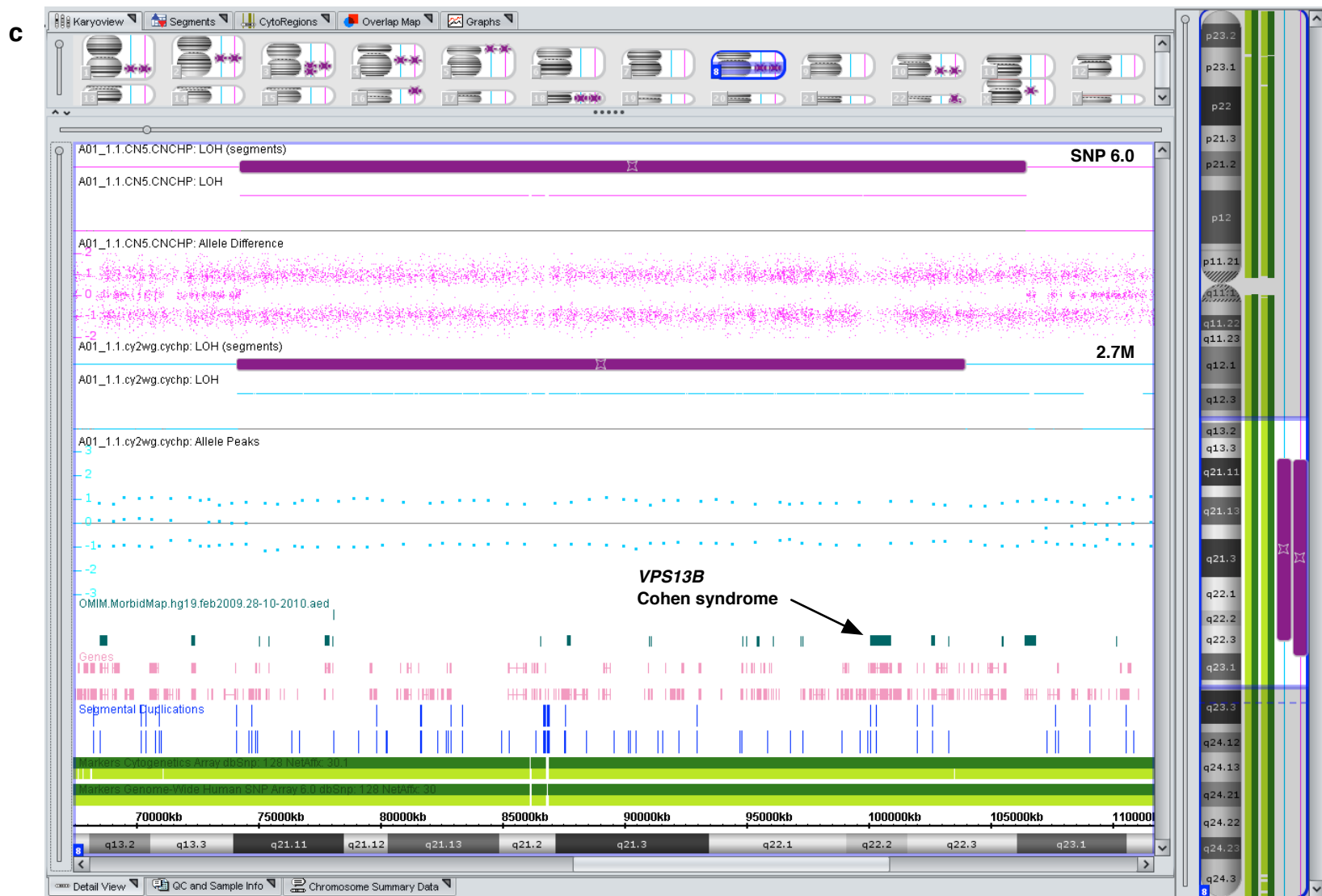

Supplement: Additional file 11: Figure S5. — Informative LCSH detected in patient 1.1, as visualized in each software. 32.2 Mb LCSH in 8q21.11q23.1, encompassing VPS13B. (A) CytoSNP. (B) Omni1. (C) SNP 6.0 and 2.7 M. [file 12920_2014_70_MOESM11_ESM.pdf]

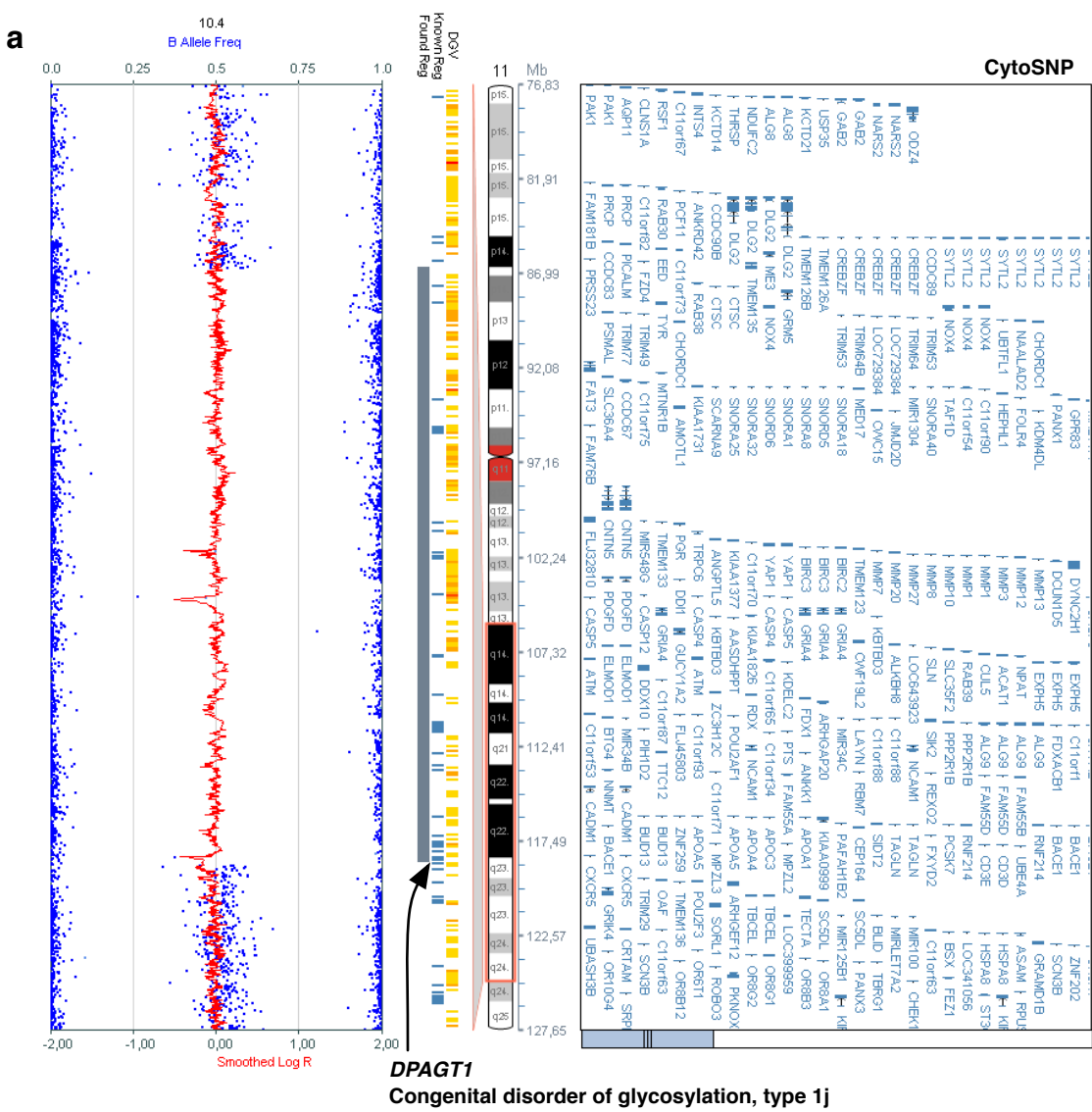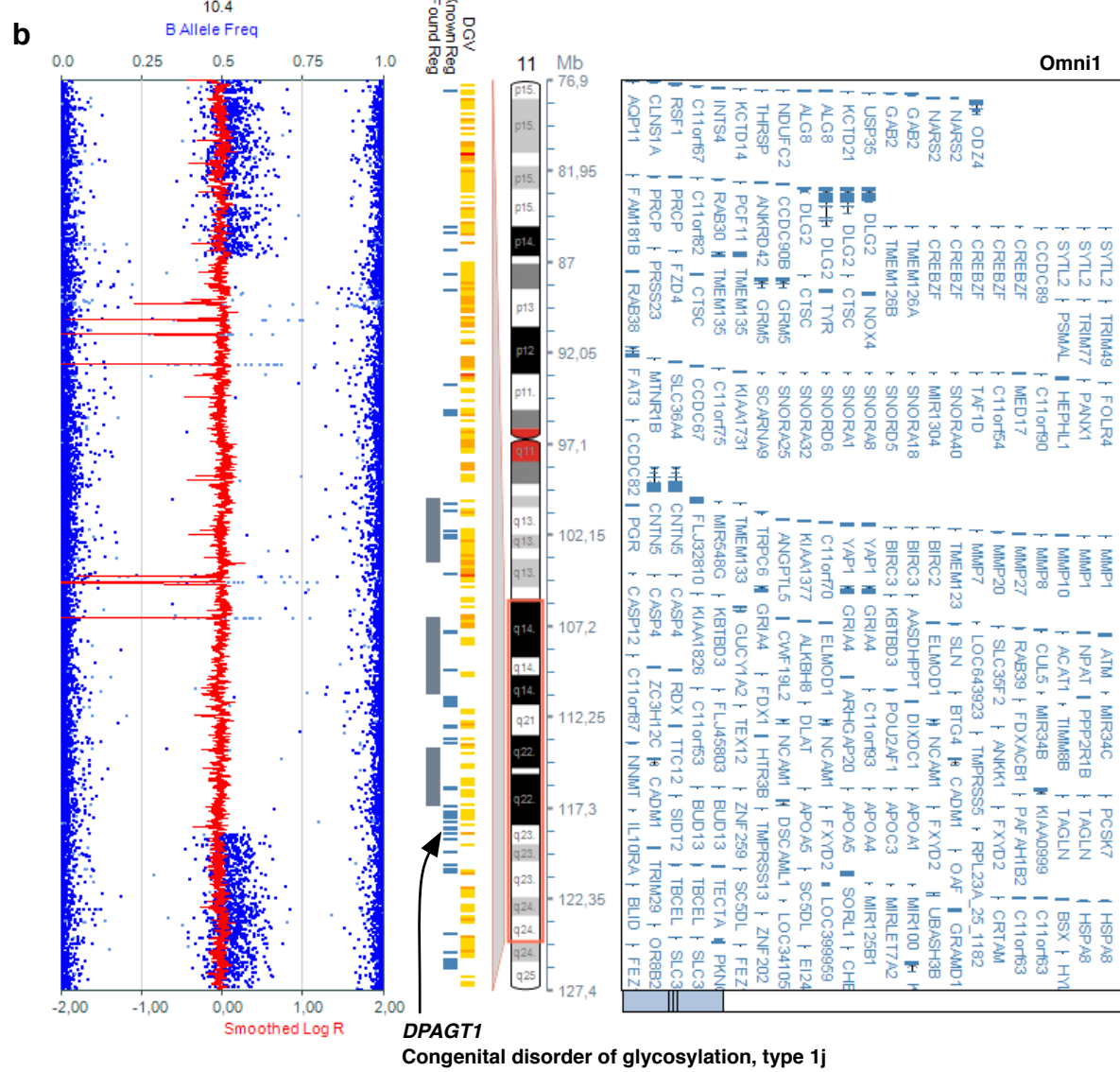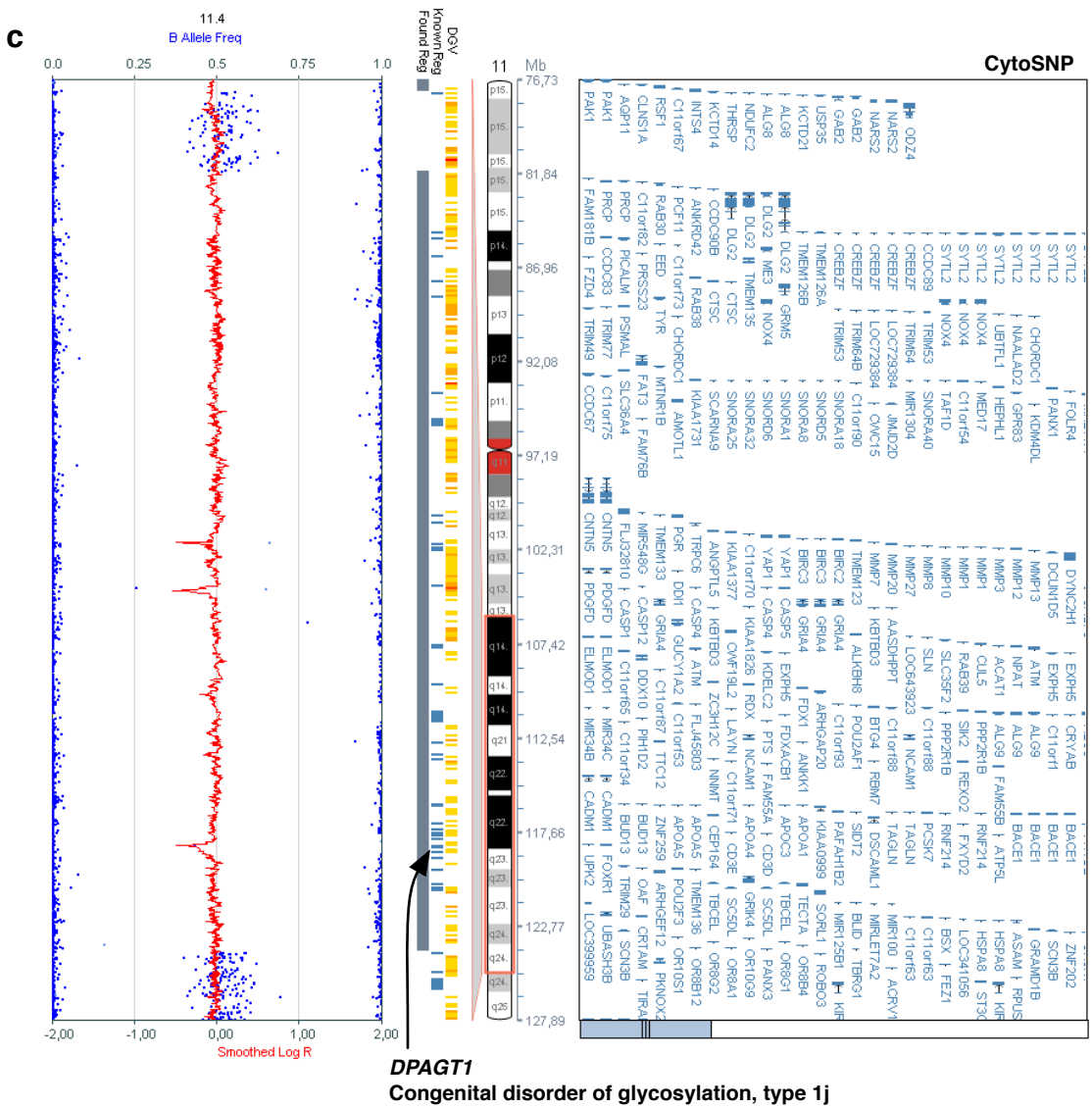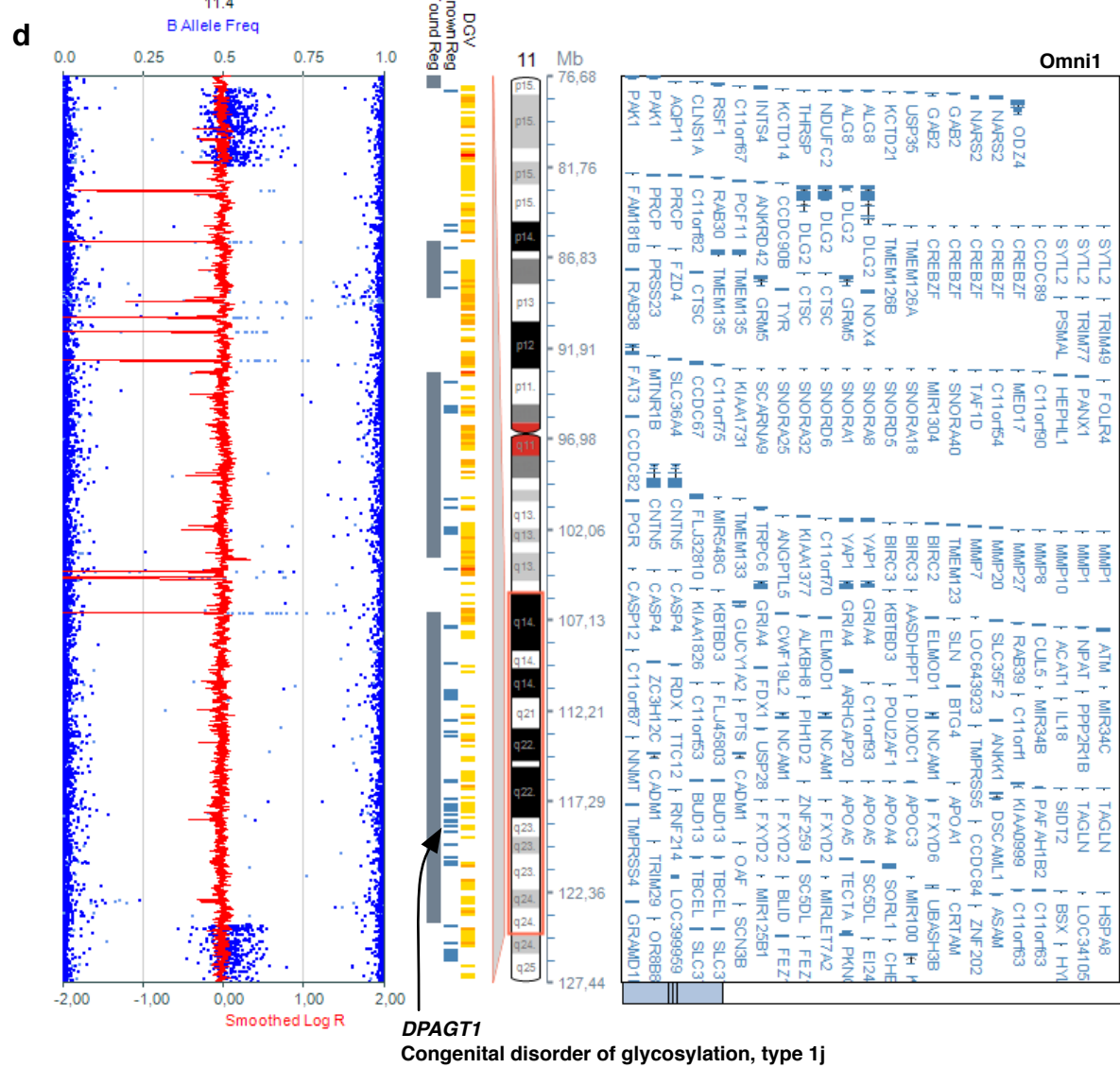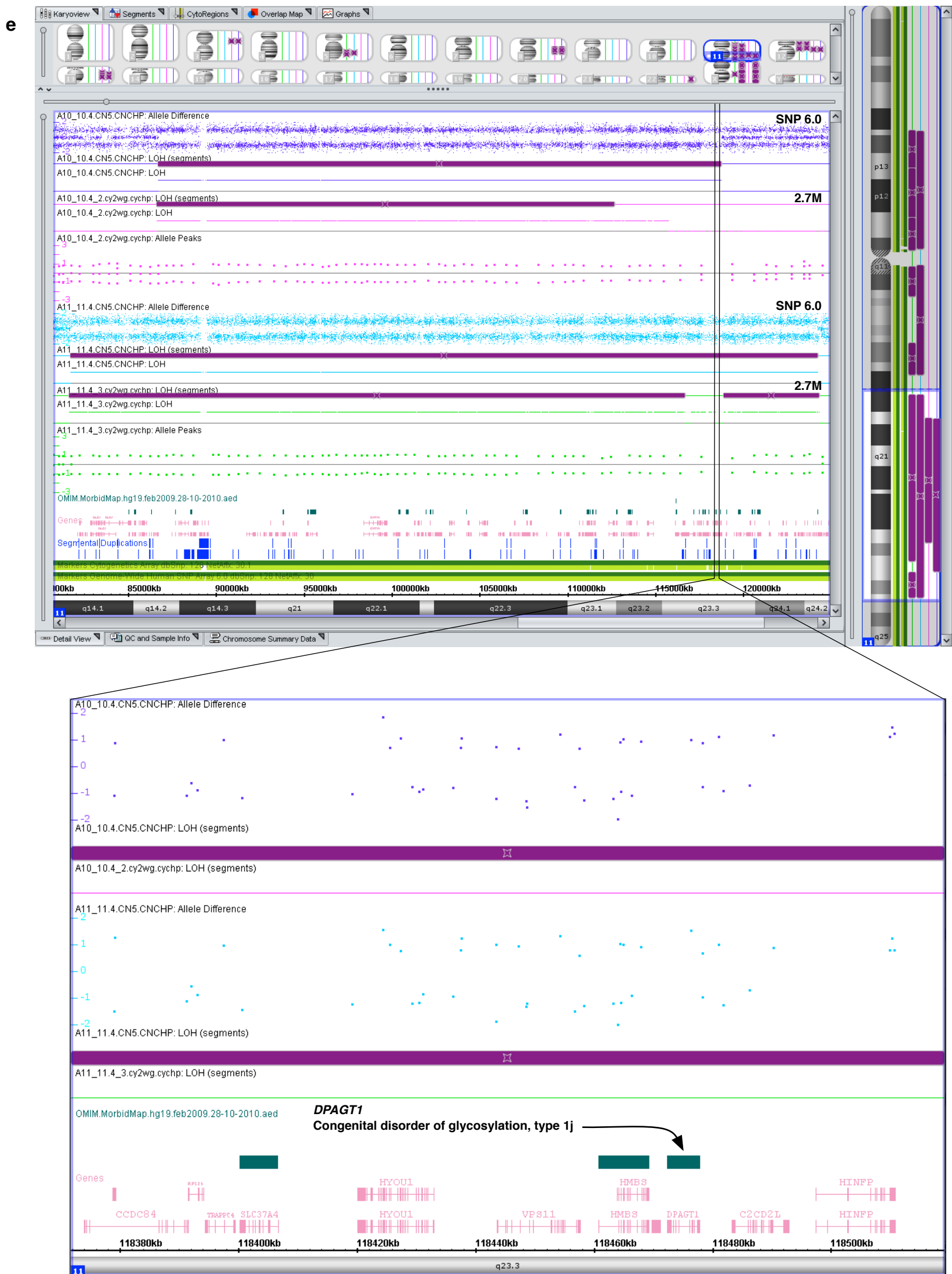

Supplement: Additional file 12: Figure S6. — Informative LCSHs detected in patients 10.4 and 11.4, as visualized in each software. 32 Mb and 42 Mb LCSHs in 11q14.1q24.2, encompassing DPAGT1. (A-B) Patient 10.4. (A) CytoSNP. (B) Omni1. (C-D) Patient 11.4. (C) CytoSNP. (D) Omni1. (E) Patient 10.4 (upper part) and 11.4 (lower part), SNP 6.0 and 2.7 M. [file 12920_2014_70_MOESM12_ESM.pdf]
