# Supplementary material for: SNP arrays: comparing diagnostic yields for four platforms in children with developmental delay
Source: BMC Med Genomics. 2014 Dec 24;7:70. doi: 10.1186/s12920-014-0070-0 (PMC4299176; doi:10.1186/s12920-014-0070-0)
Supplement: Additional file 7: Figure S3. — Pathogenic CNV detected in patient 14.5, as visualized in each software. 12q13.12q13.13 loss. (A) CytoSNP. (B) Omni1. (C) SNP 6.0 and 2.7 M. (D) CGX-12. [file 12920_2014_70_MOESM7_ESM.pdf]

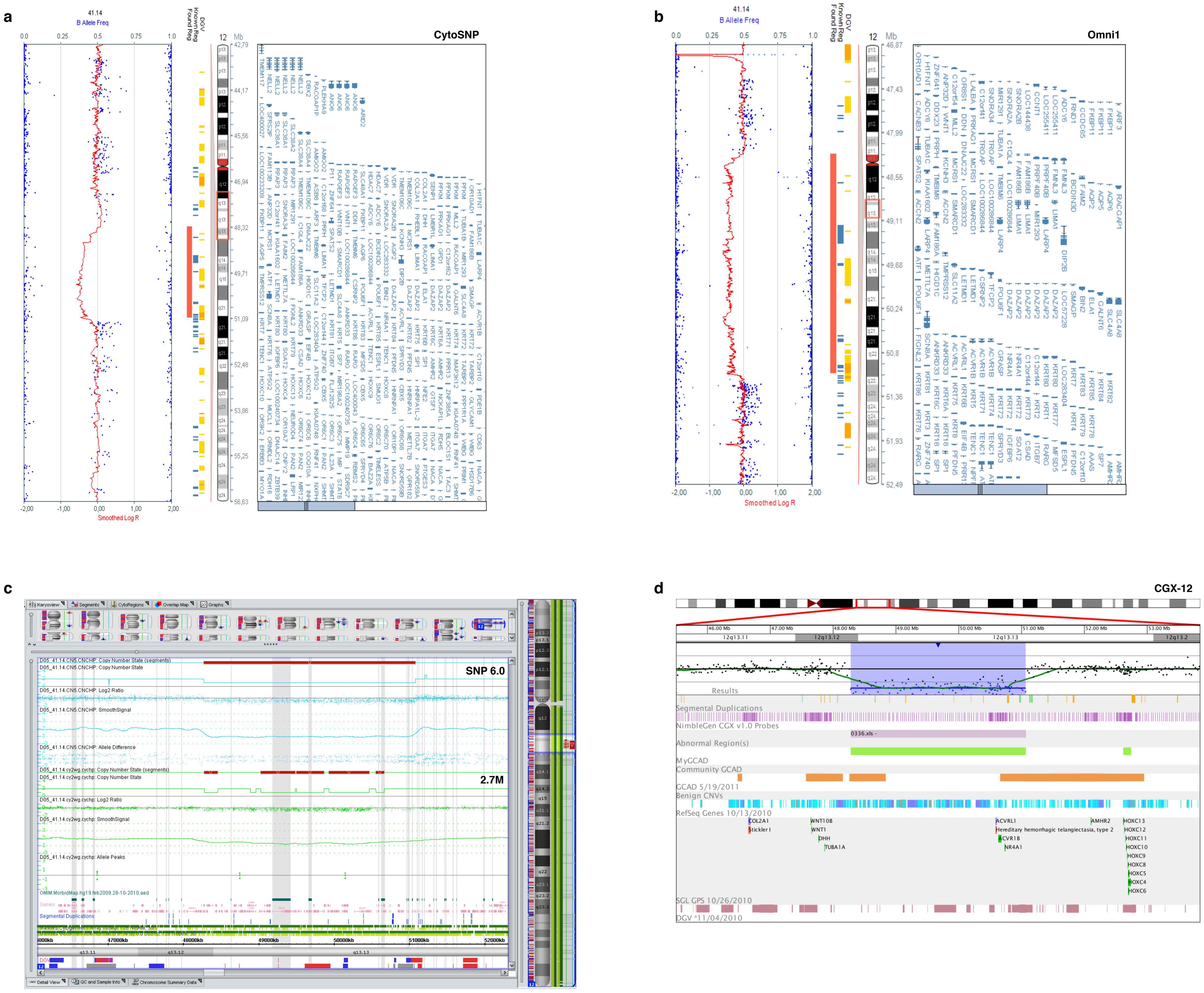
a
CytoSNP
B Allele Freq
Smoothed Log R
b
Omni1
B Allele Freq
Smoothed Log R
c
SNP 6.0
2.7M
d
CGX-12
Results
Segmental Duplications
NimbleGen CGX v1.0 Probes
Abnormal Region(s)
MyGCAD
Community GCAD
GCAD 5/19/2011
Benign CNVs
RefSeq Genes 10/13/2010
SGL GPS 10/26/2010
DGV *11/04/2010
