# Supplementary material for: SNP arrays: comparing diagnostic yields for four platforms in children with developmental delay
Source: BMC Med Genomics. 2014 Dec 24;7:70. doi: 10.1186/s12920-014-0070-0 (PMC4299176; doi:10.1186/s12920-014-0070-0)
Supplement: Additional file 8: Figure S4. — Pathogenic CNV detected in patient 44.15, as visualized in each software. 22q11.21 loss. (A) CytoSNP. (B) Omni1. (C) SNP 6.0 and 2.7 M. (D) CGX-12. [file 12920_2014_70_MOESM8_ESM.pdf]

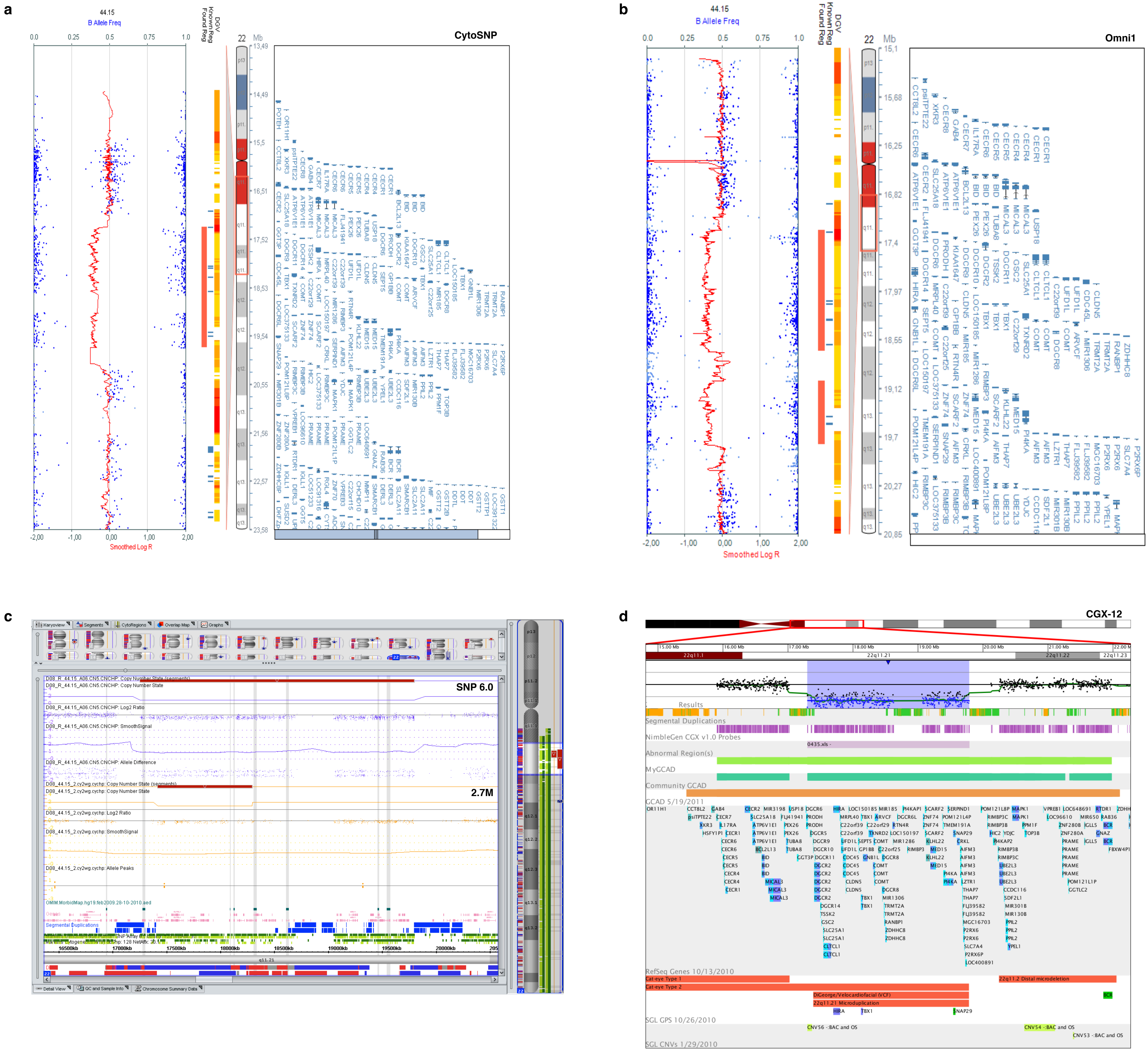
a
44.15
B Allele Freq
Smoothed Log R
DGV
Known Reg
Found Reg
CytoSNP
b
Omni1
c
SNP 6.0
2.7M
d
CGX-12
Segmental Duplications
NimbleGen CGX v1.0 Probes
Abnormal Region(s)
MyGCAD
Community GCAD
GCAD 5/19/2011
RefSeq Genes 10/13/2010
Cat-eye Type 1
Cat-eye Type 2
22q11.2 Distal microdeletion
DiGeorge/Velocardiofacial (VCF)
22q11.21 Microduplication
SGL GPS 10/26/2010
SGL CNVs 1/29/2010
