# Supplementary material for: SNP arrays: comparing diagnostic yields for four platforms in children with developmental delay
Source: BMC Med Genomics. 2014 Dec 24;7:70. doi: 10.1186/s12920-014-0070-0 (PMC4299176; doi:10.1186/s12920-014-0070-0)
Supplement: Additional file 13: Figure S7. — Informative LCSH detected in patient 26.9, as visualized in each software. 5 Mb LCSH in 2p21, encompassing LRPPRC. (A) CytoSNP. (B) Omni1. (C) SNP 6.0 and 2.7 M. [file 12920_2014_70_MOESM13_ESM.pdf]

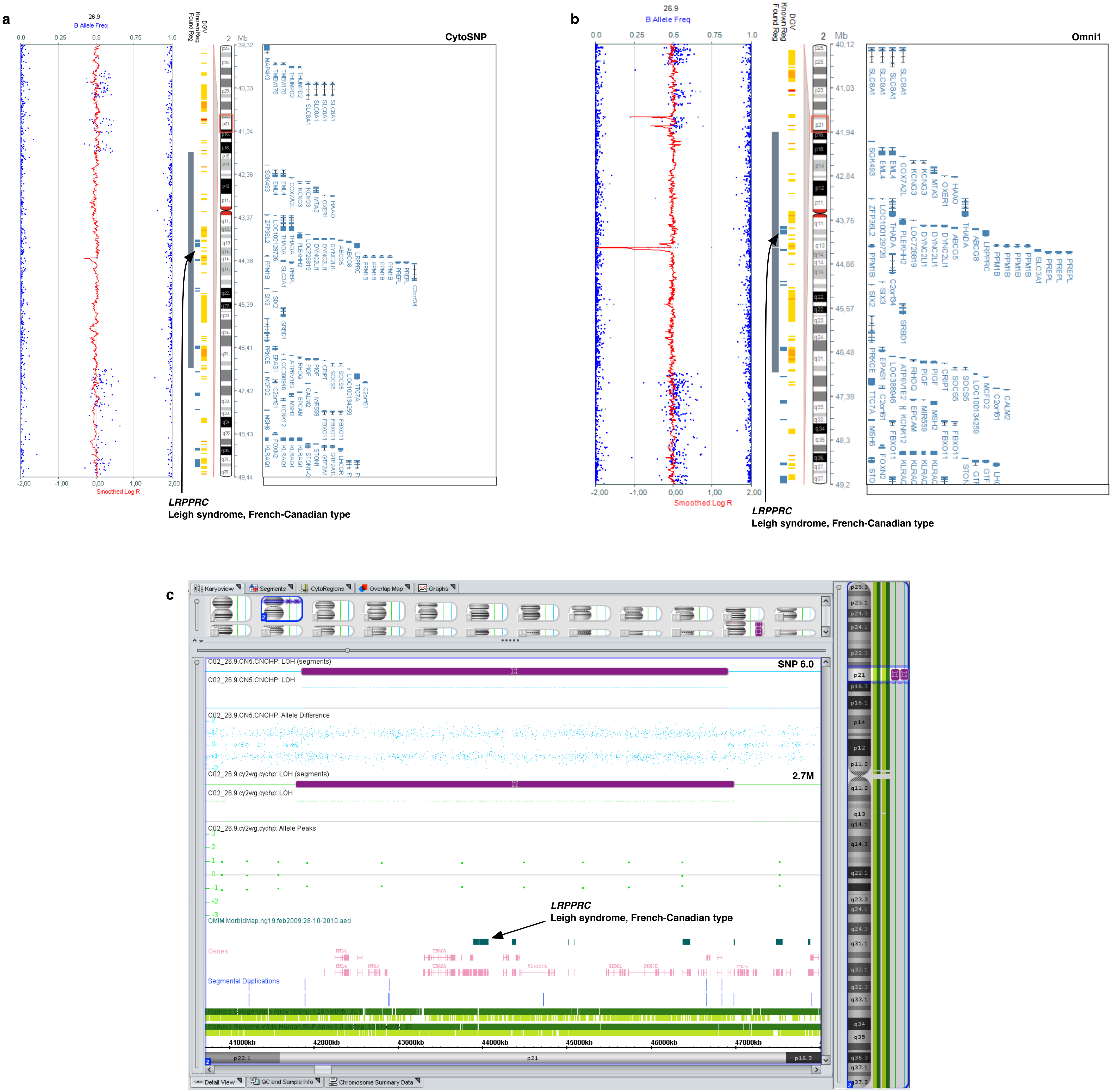
a
CytoSNP
B Allele Freq
Smoothed Log R
LRPPRC
Leigh syndrome, French-Canadian type
b
Omni1
B Allele Freq
Smoothed Log R
LRPPRC
Leigh syndrome, French-Canadian type
c
SNP 6.0
2.7M
LRPPRC
Leigh syndrome, French-Canadian type
